# Differential gene expression of immunity and inflammation genes in colorectal cancer using targeted RNA sequencing

**DOI:** 10.3389/fonc.2023.1206482

**Published:** 2023-10-05

**Authors:** Veronika Holubekova, Dusan Loderer, Marian Grendar, Peter Mikolajcik, Zuzana Kolkova, Eva Turyova, Eva Kudelova, Michal Kalman, Juraj Marcinek, Juraj Miklusica, Ludovit Laca, Zora Lasabova

**Affiliations:** ^1^ Laboratory of Genomics and Prenatal Diagnostics, Biomedical Center in Martin, Jessenius Faculty of Medicine, Comenius University in Bratislava, Martin, Slovakia; ^2^ Laboratory of Bioinformatics and Biostatistics, Biomedical Center in Martin, Jessenius Faculty of Medicine, Comenius University in Bratislava, Martin, Slovakia; ^3^ Clinic of Surgery and Transplant Center, Jessenius Faculty of Medicine in Martin, Comenius University in Bratislava, Martin University Hospital, Martin, Slovakia; ^4^ Department of Molecular Biology and Genomics, Jessenius Faculty of Medicine in Martin, Comenius University in Bratislava, Martin, Slovakia; ^5^ Department of Pathological Anatomy, Jessenius Faculty of Medicine, Comenius University in Bratislava, Martin University Hospital, Martin, Slovakia

**Keywords:** immune system, inflammation, RNA sequencing, signaling pathways, colorectal cancer, KRAS mutation, differentially expressed genes (DEG)

## Abstract

**Introduction:**

Colorectal cancer (CRC) is a heterogeneous disease caused by molecular changes, as driver mutations, gene methylations, etc., and influenced by tumor microenvironment (TME) pervaded with immune cells with both pro- and anti-tumor effects. The studying of interactions between the immune system (IS) and the TME is important for developing effective immunotherapeutic strategies for CRC. In our study, we focused on the analysis of expression profiles of inflammatory and immune-relevant genes to identify aberrant signaling pathways included in carcinogenesis, metastatic potential of tumors, and association of Kirsten rat sarcoma virus (KRAS) gene mutation.

**Methods:**

A total of 91 patients were enrolled in the study. Using NGS, differential gene expression analysis of 11 tumor samples and 11 matching non-tumor controls was carried out by applying a targeted RNA panel for inflammation and immunity genes containing 475 target genes. The obtained data were evaluated by the CLC Genomics Workbench and R library. The significantly differentially expressed genes (DEGs) were analyzed in Reactome GSA software, and some selected DEGs were used for real-time PCR validation.

**Results:**

After prioritization, the most significant differences in gene expression were shown by the genes *TNFRSF4*, *IRF7*, *IL6R*, *NR3CI*, *EIF2AK2*, *MIF*, *CCL5*, *TNFSF10*, *CCL20*, *CXCL11*, *RIPK2*, and *BLNK.* Validation analyses on 91 samples showed a correlation between RNA-seq data and qPCR for *TNFSF10*, *RIPK2*, and *BLNK* gene expression. The top differently regulated signaling pathways between the studied groups (cancer vs. control, metastatic vs. primary CRC and KRAS positive and negative CRC) belong to immune system, signal transduction, disease, gene expression, DNA repair, and programmed cell death.

**Conclusion:**

Analyzed data suggest the changes at more levels of CRC carcinogenesis, including surface receptors of epithelial or immune cells, its signal transduction pathways, programmed cell death modifications, alterations in DNA repair machinery, and cell cycle control leading to uncontrolled proliferation. This study indicates only basic molecular pathways that enabled the formation of metastatic cancer stem cells and may contribute to clarifying the function of the IS in the TME of CRC. A precise identification of signaling pathways responsible for CRC may help in the selection of personalized pharmacological treatment.

## 1 Introduction

Colorectal cancer (CRC) was the main type of cancer (17.4% of all cancer cases) in all ages of male population and the second most common cancer (14.2% of all cancer cases) in all ages of female population in Slovakia in 2020 (1). It is unflattering that Slovakia had globally the highest overall mortality rate from CRC in 2020 with age-standardized rate of 21.0 per 100,000 residents (2).

The pathogenesis of CRC is a complex interplay between genetic, epigenetic, and environmental factors. Understanding of these factors and their interactions is important for developing effective prevention and treatment strategies for this disease. A family history of CRC or colorectal polyps, familial adenomatous polyposis (FAP) or Lynch syndrome, and inflammatory bowel disease, increase a risk of CRC development in the later age (3). Lifestyle factors usually influence the development of CRC in an epigenetic or environmental mode. The well-known risk factors are overweight and/or obesity and the lack of regular physical activity, alcohol consumption, and smoking. The dietary habits in the form of low intake of fiber, fruit, and vegetables, and high intake of fat and high proportion of processed meats contribute to CRC development (3, 4).

The most cases of CRCs develop from benign polyps. These polyps often contain genetic mutations that activate oncogenes or inactivate tumor suppressor genes, leading to uncontrolled cell growth and division. The pathogenesis of CRC is characterized by genetic instability mediated through chromosomal instability, microsatellite instability (MSI), and CpG island methylator phenotype (CIMP) pathways (5).

The chromosomal instability (CIN) is observed in 65%–85% of sporadic CRCs, leading to gains or losses of large or whole proportions of chromosomes and aneuploidy in number of chromosomes, genomic amplifications, and loss of heterozygosity (LOH) (6). CIN is characterized by the inactivation of tumor-suppressor genes, as tumor protein p53 (*TP53)* and adenomatous polyposis coli (*APC*), activation of oncogenes, as *KRAS* and B-raf proto-oncogene (*BRAF*), and LOH of long arm of chromosome 18. The onset of colorectal carcinogenesis is the adenomatous stage of epithelial cells present with *APC* gene silencing and *KRAS* mutations. Subsequent inactivation of *TP53* and deletion of 18q chromosome leads to malignant transformation (7).

Microsatellite instability (MSI) presented in approximately 12%–15% of all CRCs is caused by defects in DNA mismatch repair (MMR) genes. Whereas MSI in hereditary CRC (Lynch syndrome) is caused by germline mutation in MMR genes, in the case of sporadic CRC, MSI-high (MSI-H) status is associated with CIMP-positive state resulting in hypermethylation of mutL homolog 1 (*MLH1*) and many other tumor-suppressor genes (8). Anyway, patients with MSI-H phenotype have better prognosis and higher response rate to immunotherapy (9, 10). On the other hand, approximately 85% of colorectal tumors are microsatellite stable (MSS) with worse prognosis and lower response to immunotherapy compared to MSI-H CRC (11, 12).

Metastatic CRC (mCRC) is the most common cause of death for CRC patients that have a poor 5-year survival fewer than 20%. Genomic profiling of somatic variants is very important for treatment decision and prediction of patient outcomes. Of the patients with mCRC carrying *KRAS/neuroblastoma RAS Viral oncogene Homolog (NRAS)/BRAF* wild-type tumors, 50% should be treated with combination of epidermal growth factor receptor (EGFR) monoclonal antibodies in combination with chemotherapy with few months extension in median survival. However, no effective targeted therapy is yet available for 35%–40% of patients with *KRAS/NRAS* mutation. Tumors carrying *BRAF* mutation V600E (5%–10% of mCRC) might be treated with a combination therapy with BRAF and EGFR inhibitors that may prolong the survival to 9.3 months (13).

The immune system plays a complex role in the pathogenesis and progression of colorectal cancer, with both pro- and anti-tumor effects. The studying of interactions between the IS and TME is important for developing effective immunotherapeutic strategies for CRC. In our study, we focused on the analysis of expression profiles of inflammatory and immune-relevant genes to identify aberrant signal pathways included in carcinogenesis, metastatic potential of tumors, and association of *KRAS* mutation with molecular signaling. Studying of molecular mechanisms of CRC pathogenesis and progression, immune system deregulation, and its interaction with TME could bring new possibilities in diagnosis, treatment strategies, and identification new potential immunotherapy targets.

## 2 Methods

### 2.1 Patients, clinical tissue samples, and RNA extraction

In total, 91 CRC patients underwent resection of CRC at Clinic of General, Visceral and Transplant Surgery, University Hospital Martin. Samples were collected from cancer (n=91) and adjacent (n=71) tissues in collaboration with Department of Pathological Anatomy, University Hospital Martin. The patient characteristics are presented in **Table 1**. The primary (n=52) and metastatic tumors (n=30) with metastases in the liver were evaluated by the experienced pathologist, and tumor tissue surgical excisions were immersed into Dulbecco’s modified Eagle’s medium (DMEM), penicillin/streptomycin, and 10% of fetal bovine serum and stored at 4°C. The samples treated this way were delivered to the Department of Molecular Biology and Genomics and transferred to RNA later and stored at −80°C until RNA extraction. Other sample criterion of the tissue collection above was the presence (n=34) and absence (n=49) of KRAS mutation. Tissues were homogenized, and total RNA was extracted using RNeasy Mini Kit (Qiagen, Hilden, Germany) according to the manufacturer’s instructions. The eluted RNA was then stored at −80°C until reverse transcription reaction or RNA-seq libraries preparation.

**Table 1:** Clinicopathological characteristics of patients included in the study.

Patient characteristics		Number of patients (n)
**Average age**		67.5 (SD ± 9.5)
**BMI**		27.9 (SD ± 4.7)
**Gender**	Female	39 (42.9%)
	Male	52 (57.1%)
**Grade**	G1	15 (16.5%)
	G2	41 (45%)
	G3	13 (14.3%)
	N/A	22 (24.2%)
**T stage**	T1	3 (3.3%)
	T2	15 (16.5%)
	T3	49 (53.8%)
	T4	16 (17.6%)
	N/A	8 (8.8%)
**N stage**	N0	39 (42.9%)
	N1	29 (31.9%)
	N2	14 (15.3%)
	N/A	9 (9.9%)
**M stage**	M0	41 (45%)
	M1	38 (41.8%)
	N/A	12 (13.2%)
**Clinical stage**	I	9 (9.9%)
	II	20 (22%)
	III	15 (16.5%)
	IV	35 (38.4%)
	N/A	12 (13.2%)
**Tumor type**	Primary tumor	50 (54.9%)
	Tumor with liver metastases	32 (35.2%)
	N/A	9 (9.9%)
**KRAS gene mutation**	present	34 (37.4%)
	absent	49 (53.8%)
	N/A	8 (8.8%)

CRC, colorectal cancer; BMI, body mass index; N/A, not applicable; T, tumor size; N, lymph nodes positive for tumor; M, metastasized cancer; KRAS, Kirsten rat sarcoma virus.

### 2.2 RNA sequencing of inflammation and immunity gene expression

Using next generation RNA sequencing, a differential gene expression analysis of 11 tumor and 11 matching non-tumor tissues was carried out by applying a targeted RNA panel for inflammation and immunity genes (Qiagen, Germany) containing 475 target genes and 25 reference normalization genes. Extracted RNA was quantified by fluorometric quantitation (Qubit 3.0, Invitrogen, USA), and RNA integrity was checked by 2100 Bioanalyzer (Agilent). RNA-seq libraries for CRC and adjacent tissue were prepared using QIAseq Targeted RNA Human Inflammation & Immunity Transcriptome (Qiagen). The panel is designed for detection of DEGs for pro- or antiapoptotic genes, cytokines, chemokines, growth factors and their receptors, and transcription factors performing various functions of the immune system. cDNA libraries were prepared from 450 ng of RNA and assigned with Unique Molecular Indexes (UMIs). Quality control was performed by Bioanalyzer, and libraries were sequenced using single-end reads, 1× 150 bp on MiSeq (Illumina, USA), to a depth of 5 million reads. Particular investigation of the role of significantly deregulated DEGs has been provided by NcPath online analysis tool, which allows visualization and enrichment for non-coding RNA and KEGG signaling pathways in humans to elucidate the physiological and pathological processes in CRC (14).

### 2.3 Experimental validation of RNA-seq data by qPCR

Six differentially expressed genes were selected for validation using quantitative real-time PCR (qPCR). The 500 ng of sample RNA and reference RNA (Universal Human Reference RNA, Invitrogen) was reversely transcribed to cDNA using High-Capacity cDNA Reverse Transcription Kit with RNase Inhibitor (Applied Biosystems). In the less concentrated samples, the initial concentration of 250 ng was processed to reverse transcription (RT). No Enzyme Control (NEC) was also included in each series of transcription. The temperature steps of RT include incubation at 25°C for 10 min, RT at 37°C for 120 min, and enzyme inactivation at 85°C for 5 min. Samples were stored at −20°C until further use.

The initial step of relative quantification (RQ) includes 10-fold serial dilutions of reference RNA from 25 ng to 2.5pg. The six target assays, namely, receptor interacting serine/threonine kinase 2 (Applied Biosystems, RIPK2, Hs01572686_m1, FAM-MGB), interleukin 6 receptor (IL6R, Hs01075664_m1, FAM-MGB), TNF superfamily member 10 (TNFSF10, Hs00921974_m1, VIC-MGB), MHC class I polypeptide-related sequence B (MICB, Hs00792952_m1, FAM-MGB), C-C motif chemokine receptor 4 (CCR4, Hs01396342_m1, FAM-MGB), and B-cell linker (BLNK, HS00179459_m1, VIC-MGB), and two housekeeping genes, actin beta (ACTB, HS99999903_m1, VIC-MGB) and glyceraldehyde-3-phosphate dehydrogenase (GAPDH, Hs99999905_m1, VIC-MGB), were tested. Each duplex reaction was run with TaqMan™ Fast Advanced Master Mix (Applied Biosystems) in the total volume of 20 μl with thermal cycling conditions, as incubation at 50°C for 2 min, polymerase activation at 95°C for 2 min, and 40 cycles with denaturation at 95°C for 3 s and annealing/extension at 60°C for 30 s in the instrument 7500 Fast Real-Time PCR System (Applied Biosystems). Standard curves were created for each tested assay from dilution series in the 7500 instrument software, and pairs of assays labeled with FAM and VIC have been chosen for duplex reactions (IL6R+BLNK, MICB+TNFSF10, RIPK2+GAPDH, and CCR4+ACTB). Data are not present in the study.

For validation of RNA sequencing by RQ, 91 tumors and 71 adjacent tissues were selected. Samples were analyzed in duplicates for each duplex reaction, and all pipetting steps were performed on BRAVO Liquid Handling Station (Agilent) to minimalize subjective pipette handling bias. The results were analyzed in the instrument software with unique threshold setting and cycle threshold (Ct value)calculation.

### 2.4 Pathway analysis using Reactome

RNA sequencing data from immune and inflammation panel were processed in inter-run normalization to eliminate technical differences between four runs. The corrected data were analyzed in Reactome data online analysis tool. Reactome (15), as online data analysis tool, was used to map the biological pathways influenced by DEGs identified in the study. The batch-corrected RNA-seq data of four experiments (six samples per run, including tumor and adjacent tissue) have been submitted to gene expression analysis. The PADOG (16) (weighted gene set analysis method that downweighs genes that are present in many pathways) method has been applied to RNA-seq normalized data, recorded with annotations as cancer or control, primary or metastatic tumor or control, and KRAS mutation positive or negative. Overall, the DEGs from the RNA-seq study of 11 CRC and 11 adjacent tissues have been enriched in 1,163 biological pathways, and 461 DEGs were identified. The selected pathways with a p-value for entity <0.05 have been considered as statistically significant.

### 2.5 Bioinformatics processing and data analysis

#### 2.5.1 RNA-seq data evaluation

Sequencing data (fastq files) were imported into CLC Genomics Workbench (GW) v. 21.0.4 and processed by the RNA-seq analysis pipeline with the default settings. The report, generated by the pipeline, was utilized for obtaining information on the quality control. Transcripts per million (TPM), which are normalized for sequencing depth so their values are comparable between samples, were used in data analysis outside CLC. Differential expression analysis was performed using differential expression for RNA-seq pipeline with meta-data specifying both groups (e.g., case vs. control) and run, so that the batch correction was included into the computation of the average fold change (averaged over the “case” samples). A heatmap was created using Create Heat Map for RNA-Seq tool. In order to obtain a set of differentially expressed genes (DEGs), we used the elastic network Machine Learning algorithm. The TPM data were first subjected to batch correction by the scBatch method (17), scaled and then fed to the elastic network algorithm (18) (see, e.g., (19), for an overview of ML in RNA-seq), using R (20), ver. 4.0.5. Genes with positive variable importance were selected as DEGs. CLC was used to create heatmap for DEGs.

#### 2.5.2 RT-PCR

Fold change (FC) was computed using the standard formula (i.e., 2^−ΔΔCt^). The data on FC and log2(FC) were explored in R, by means of boxplot overlaid with swarmplot. Distribution of log2(FC) was assessed by the quantile–quantile plot with the 95% confidence band constructed by bootstrap. Since the distribution of log2(FC) was either Gaussian, close to Gaussian, or symmetric, we used the Wilcoxon test to test the null hypothesis that the population median (point of symmetry) of log2(FC) is 0. p-Values were not subjected to a correction for multiple hypothesis testing. In order to compare FC obtained by RNA-seq with those from RT-PCR, the FC of RT-PCR was averaged over the “case” samples (patients in the FC of patients vs. controls; metastatic patients in the case of FC of metastasis vs. primary tumor; KRAS+ for FC of KRAS+ vs. KRAS−). A correlation between RNA-seq and qPCR data was calculated by the FC from RNA-seq, which is in the averaged form produced by CLC GW, and values were cross-plotted against the average FC from RT-PCR. A 45° line was added to the plot, to facilitate comparison of the methods.

## 3 Results

### 3.1 Evaluation of RNA-sequencing data

We analyzed the differential gene expression of immunity and inflammation genes (475 genes) in the CRC (n=11) and matching adjacent (n=11) tissues of 11 patients. The other inclusion criterion to the study was the condition of KRAS gene that is frequently mutated in CRC. From the analyzed samples, primary CRC (prim CRC) without mutation in KRAS was present in two samples (n=2), and primary CRC with a mutation in the KRAS gene was identified in three patients (n=3). Other three samples were from CRC patients with metastases (mtsCRC) in the liver, and wild-type allele of KRAS gene (n=3) and other three mtsCRC had also mutation in KRAS gene (n=3).

**Figure 1:**
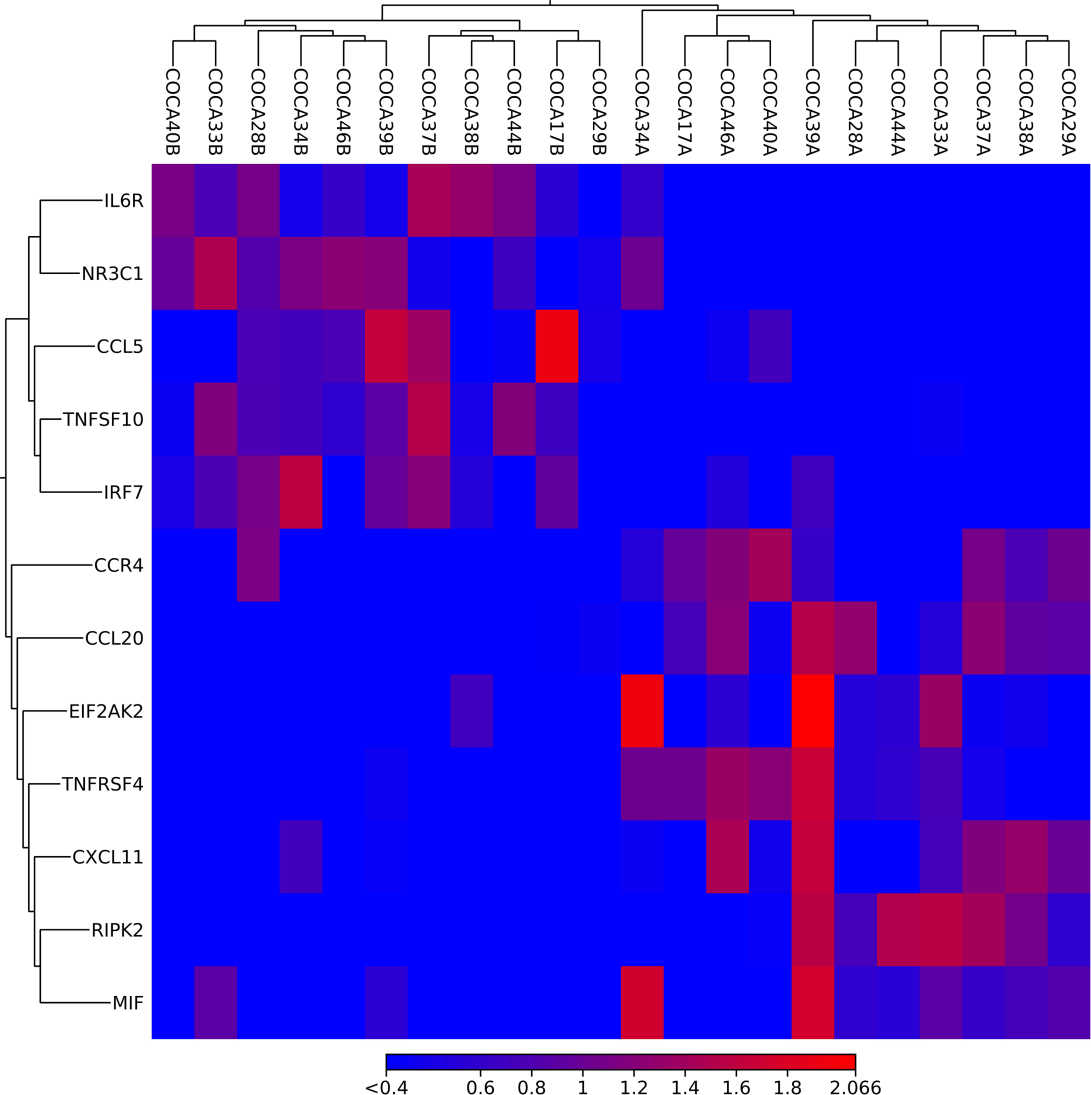
A heatmap created in CLC on based on the importance of DEGs. The upper horizontal axis represents sample marks colorectal (CO) cancer (CA) ending with A (cancer tissue) or B (adjacent tissue). The vertical axis presents the names of DEGs. The red color represents upregulation, and blue color reflects downregulation of DEGs.

After the inter-run normalization and data evaluation in CLC, DEGs were selected based on positive variable importance (**Table 2**), and a heatmap was generated (**Figure 1**). Overall, mRNA expression of 478 differentially expressed genes were found after alignment. From the designed set of DEGs, MPL, GDC_Cont, IL3, IL9, IFNW1, IFNA4, IFNA14, IFNA6, IFNA1, and IL25 expression was not found in CRC and in adjacent tissue.

**Table 2:** Statistical analysis of batch-corrected data and feature selection revealed top 12 significant DEGs.

	Gene	Importance
1	TNFRSF4	0.915
2	IRF7	0.720
3	IL6R	0.678
4	NR3C1	0.501
5	EIF2AK2	0.434
6	MIF	0.264
7	CCL5	0.209
8	TNFSF10	0.123
9	CCL20	0.114
10	CXCL11	0.112
11	RIPK2	0.095
12	CCR4	0.005

A pathway analysis revealed that the most significantly expressed DEGs are enriched in cytokine (hsa-04061) and chemokine signaling pathways (hsa04062), and in inflammatory diseases (hsa05323 and hsa05417). Some DEGs are involved in pathways directly associated with diseases of the digestive tract (hsa04672 and hsa05120) or identified in cancer (hsa04668, hsa05200, and hsa05203). Moreover, some DEGs are enriched in infectious viral and bacterial diseases (hsa05163, hsa05134, hsa05171, hsa05131, hsa05133, hsa05135, hsa05132, hsa05130, hsa05162, hsa05164, and hsa05167) and in signaling pathways (hsa04620 and hsa04217) that are important in the immune defense against viral, bacterial, fungal pathogens, and parasites (21) and in mediating of inflammation by inducing type 1 interferon (IFN) production (hsa04623) (22).

**Figure 2:**
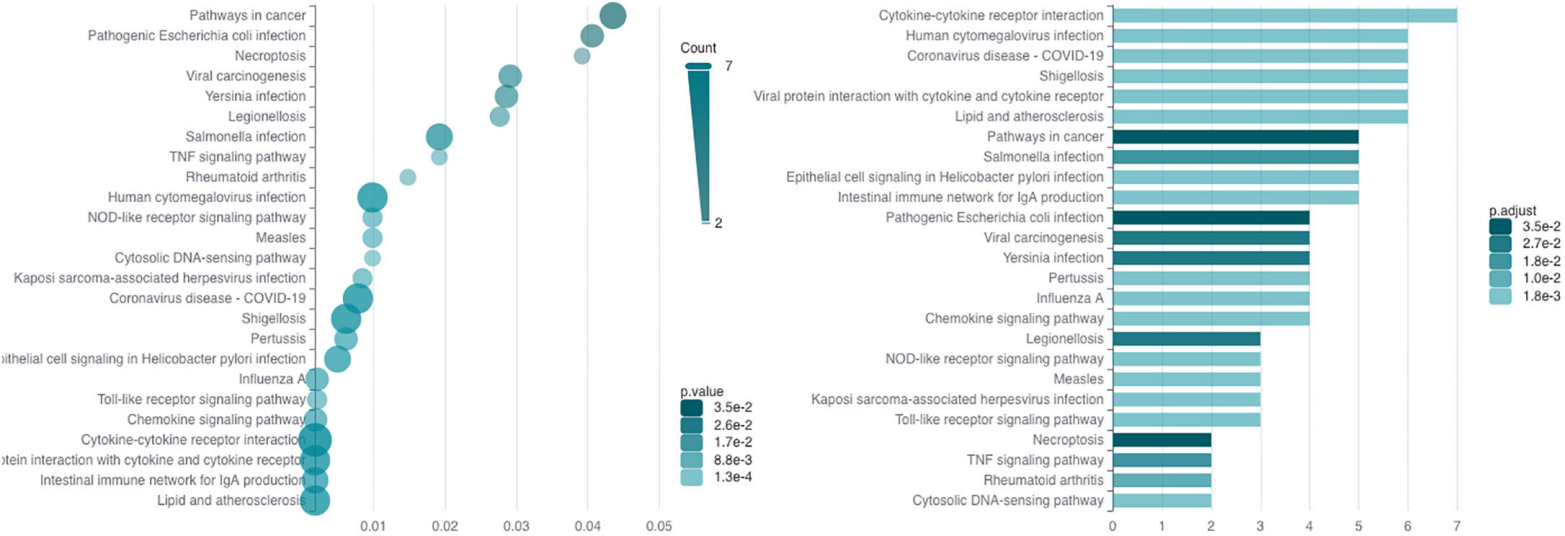
The result of significantly DEG enrichment in KEGG signaling pathways. The graph on the left side represents the p-values for DEG enrichment in KEGG pathways, and the graph on the right represents the number DEGs enriched in the pathways; the intensity of green color means the significance of the value.

The signaling pathways are listed in **Supplementary Table 1** and displayed in **Figure 2**.

### 3.2 Validation of DEGs on a larger cohort of CRC tissues

From the prioritized and other genes, six DEGs (BLNK, CCR4, ILR6, MICB, RIPK2, and TNFSF10) and two housekeeping genes (ACTB and GAPDH) were chosen to validate study results on a larger cohort of 162 tissue samples, including 91 CRC tissues and 71 adjacent tissues. Statistical analysis was performed to determine the fold changes between groups, as cancer/control tissues, metastatic and primary tissues, and KRAS-positive/KRAS-negative tissue. The corresponding p-values are presented in **Table 3**.

**Table 3:** The qPCR results of selected DEG fold change analysis between the above-mentioned sample groups with corresponding p-values.

gene	Cancer vs. control tissue	Metastatic vs. primary CRC	KRAS-positive vs. KRAS-negative CRC
	p-value	p-value	p-value
BLNK	<0.001	0.517	0.055
CCR4	0.278	0.550	0.281
ILR6	<0.001	0.107	1.000
MICB	0.044	0.977	0.050
RIPK2	<0.001	0.318	0.723
TNFSF10	<0.001	0.318	0.072

CRC, colorectal cancer; KRAS, Kirsten rat sarcoma virus mutation positive; BLNK, B-cell linker; CCR4, C-C Motif Chemokine Receptor 4; ILR6, interleukin 6 (IL6) receptor complex; MICB, MHC class I polypeptide-related sequence B; RIPK2, receptor interacting serine/threonine kinase 2; TNFSF10, TNF superfamily member 10.

**Figure 3:**
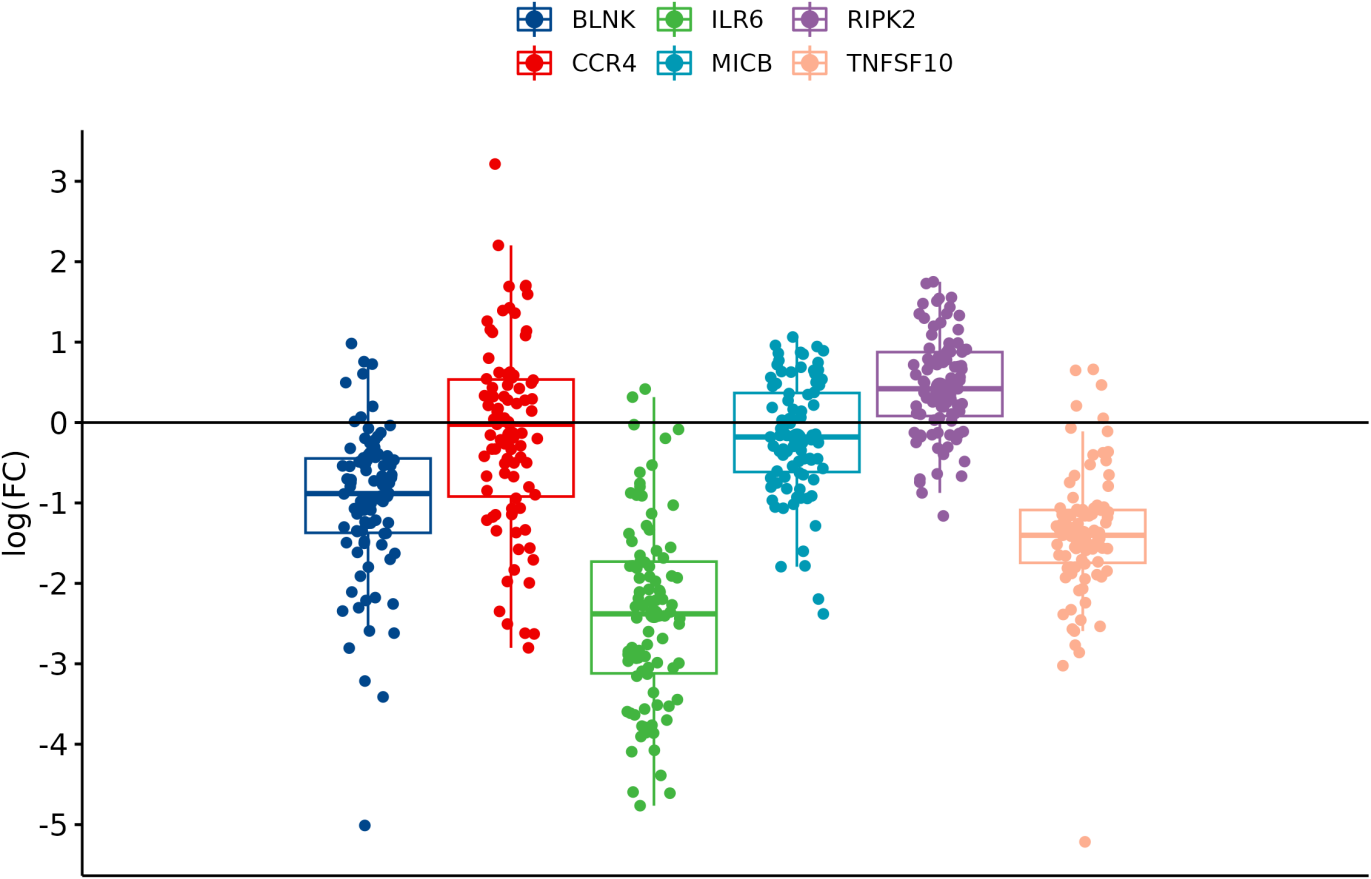
Boxplots showing fold change difference of DEGs in CRC tissue compared to adjacent tissue that was investigated on validation cohort.

**Figure 4:**
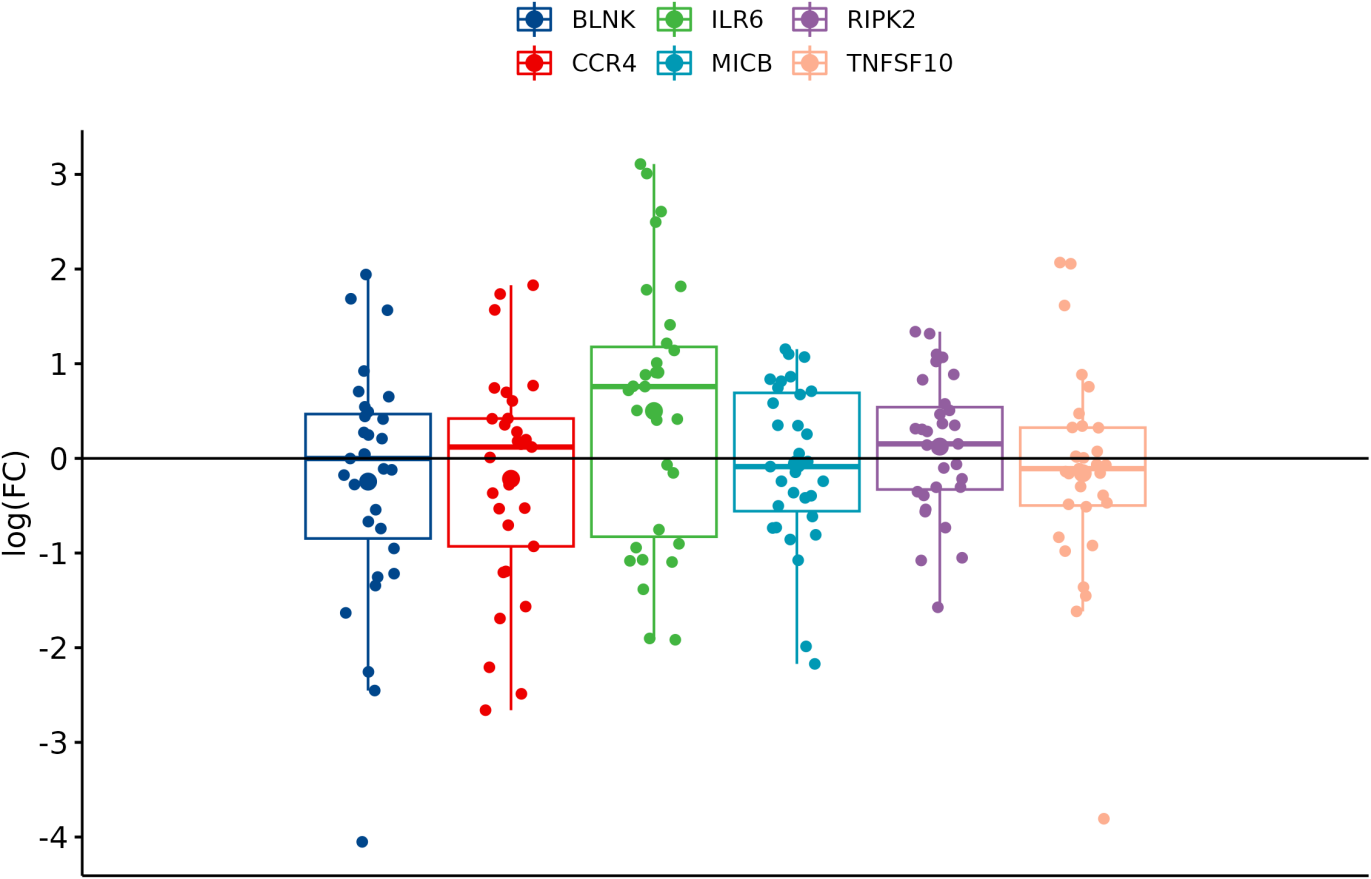
Boxplots showing fold change difference in DEGs in the metastatic CRC tissue compared to primary tumor that was investigated on validation cohort.

**Figure 5:**
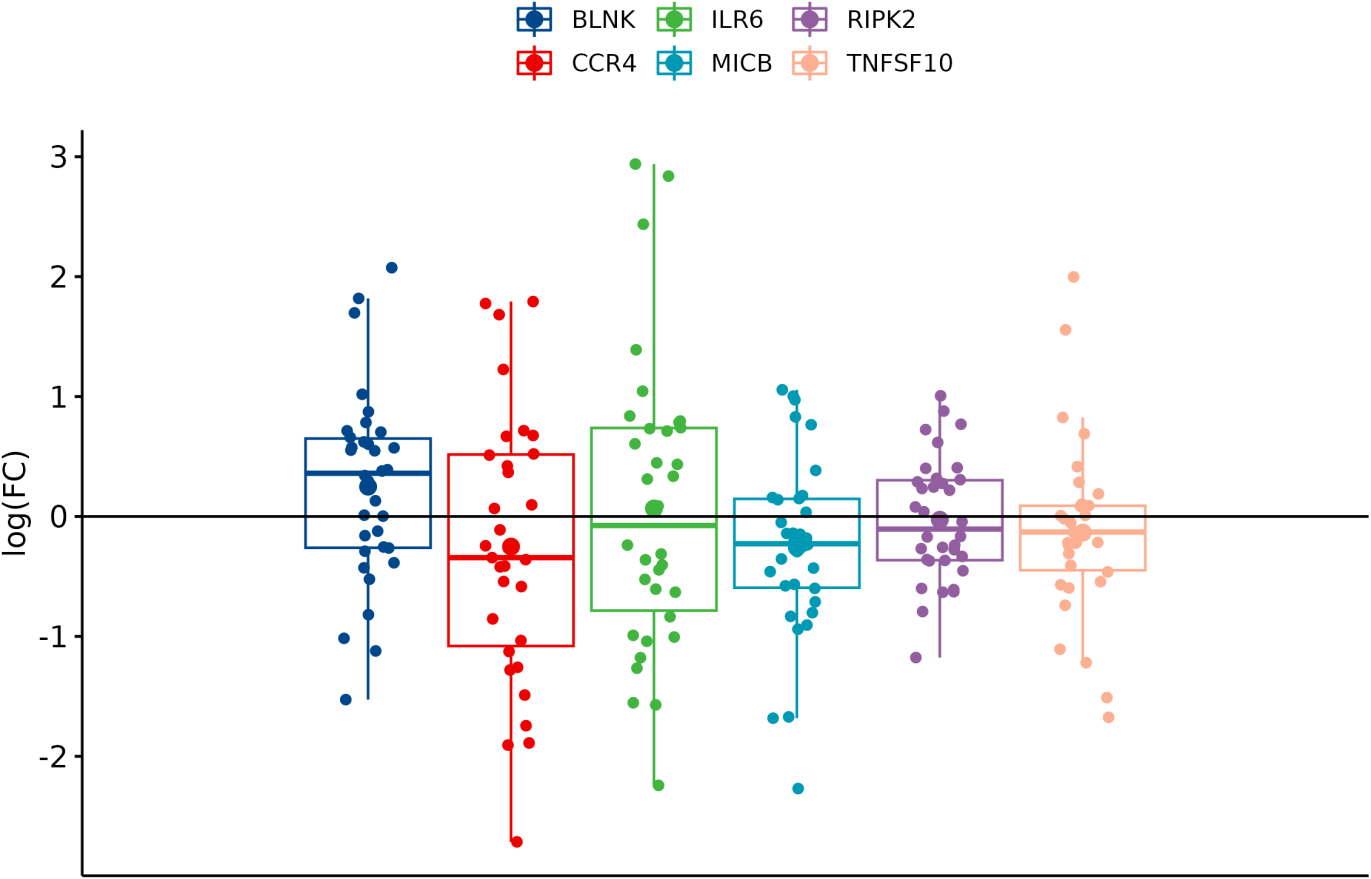
Boxplots showing fold change difference in DEGs in KRAS-positive versus KRAS-negative CRC tissue that was investigated on validation cohort.

In comparison between cancer and adjacent control tissue, all the selected DEGs have been significantly changed in cancer CRC tissues, in exception to CCR4. The remaining DEGs, such as BLNK, IL6R, MICB, and TNFSF10, were identified with significantly decreased expression, and RIPK2 was identified with significantly increased expression (**Figure 3**). In the group of metastatic vs. primary CRC tissue, no significant differences in selected DEGs were found. BLNK expression was equal in metastatic cancer tissues when compared to the control tissue. DEG expression of CCR4, ILR6, and RIPK2 was increased, and the expression of MICB and TNFSF10 was decreased in metastatic tissues (**Figure 4**). KRAS-positive tissues revealed weakly significant upregulation of BLNK and downregulation of MICB and TNFSF10 expression when compared to KRAS-negative cancer tissue. DEG expression in KRAS-positive tissues was equal for ILR6 and decreased for CCR4 and RIKP compared to KRAS-negative FCs (**Figure 5**). The complete results of bioinformatical analysis of qPCR fold changes are added in **Supplementary Data Sheet 1**.

### 3.3 A correlation between qPCR and RNA-sequencing data

**Figure 6:**
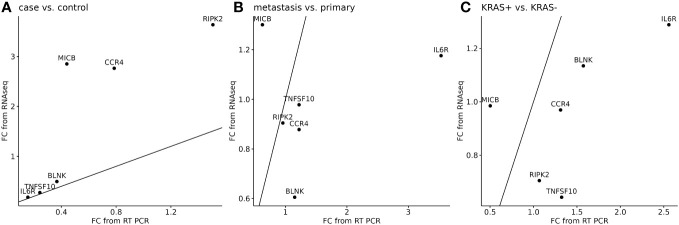
Correlation of RNA-seq data (n=11) to the matching samples analyzed by qPCR (n=11) for six selected DEGs. A separate analysis was performed to cancer or control samples **(A)**, metastatic or primary tumor **(B)**, and KRAS-positive or KRAS-negative tumors **(C)**. 45° line was added to the plot to facilitate comparison of the methods.

**Figure 7:**
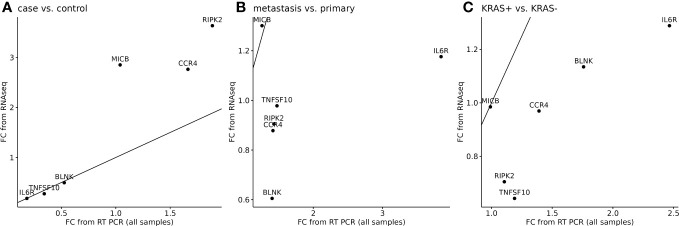
Correlation of RNA-seq data to all samples analyzed by qPCR for six selected DEGs. A separate analysis was performed to cancer tissues (n=91) or control adjacent tissues (n=71) samples **(A)**, metastatic or primary tumor **(B)**, and KRAS-positive or KRAS-negative tumors **(C)**. 45° line was added to the plot to facilitate comparison of the methods.

The next analysis was performed to see a correlation between two widely used methods for measuring RNA expression. We have calculated the relative gene expression (fold change, FC) for selected genes analyzed by qPCR. In RNA-seq data, the average FC was calculated from sample-by-sample computed FC for each gene and was correlated to the average FC of qPCR data of selected genes. To quantify potential discrepancies between RNA-seq and qPCR, the calculated gene expression fold changes of 11 RNA-seq samples were compared to the same samples analyzed in qPCR (**Figure 6**) and to all samples analyzed by qPCR (**Figure 7**). The main groups in this correlation were cancer vs. control, metastatic vs. primary cancer, and KRAS-positive vs. KRAS-negative tumors. High fold change correlation for IL6R, TNFSF10, and BLNK DEGs was found between the matching samples and all CRC tissue samples in cancer vs. control correlation. The other two groups, namely, primary or metastatic cancer and KRAS positive or negative, have a weak correlation between RNA-seq and qPCR data for each selected DEG. The complete correlation data are attached in **Supplementary Data Sheet 2**.

### 3.4 Analysis of biological pathways by Reactome v83

RNA sequencing data from immune and inflammation panel were processed in inter-run normalization to eliminate technical differences between four runs. The corrected data were analyzed in Reactome data online analysis tool. We used PADOG data analysis algorithm that is Reactome recommended as classical gene set analysis approach. Overall, the DEGs from the RNA-seq study of 11 CRC and 11 adjacent tissues have been enriched in 1,163 biological pathways, and 461 DEGs were identified. We have applied filtering of the pathways by an adjusted p-value ≤0.05 that have been considered as significantly regulated by DEGs.

#### 3.4.1 A comparison of biological signaling pathways between cancer and control adjacent tissues

This part of the study was performed to see which signaling pathways were altered in CRC tissue against adjacent control tissue. The DEGs from the RNA-seq study of 11 CRC and adjacent tissues have been enriched in 66 biological pathways (**Supplementary Image 1**). The top 10 up- and downregulated DEGs are presented in **Table 4**. In summary, the Reactome analysis identified seven DEGs (MIF, RIPK2, IRF7, IL6R, NR3C1, TNFSF10, and CCL5) that were also identified as important in RNA-seq analysis, and three of them were used in RNA-seq validation on a larger cohort.

**Table 4:** Top 10 up- and downregulated DEGs identified in CRC compared to the control tissue; logFC and p-values were estimated by the Reactome v83.

Identifier	Regulation	LogFC	p-value	Identifier	Regulation	LogFC	p-value
**ELK1**	Up	369.89	<0.001	**IRF7**	Down	−1,359.67	<0.001
**CDK2**	Up	560.34	<0.001	**IL6R**	Down	−364.34	0.003
**MYC**	Up	2001.07	<0.001	**IFIT2**	Down	−412.10	0.003
**GPI**	Up	6,285.67	<0.001	**NFKBIA**	Down	−13,101.91	0.004
**MIF**	Up	34,409.42	<0.001	**NR3C1**	Down	−842.99	0.006
**RIPK2**	Up	597.92	<0.001	**TP53INP1**	Down	−1,178.21	0.006
**CD47**	Up	2,780.45	<0.001	**TNFSF10**	Down	−2,888.22	0.008
**IRF2BP1**	Up	142.78	<0.001	**CCL5**	Down	−1,535.82	0.012
**CXCL3**	Up	1,612.90	<0.001	**ACKR2**	Down	−115.06	0.014
**PRKRA**	Up	1,115.71	<0.001	**MAF**	Down	−1,606.18	0.015

Subsequently, we have analyzed 66 differentially regulated biological pathways related to signal transduction and overactivity of tyrosine kinases frequently occurring in cancer tissues. Presumably, the main finding in this part of the study is a gene expression–transcription, especially changes in signaling pathways associated with TP53 activity. The changes between cancer and control adjacent tissue were also identified in disease signaling pathways, DNA repair, and programmed cell death, especially in the downregulation of apoptosis and defects in cell cycle signaling and developmental biology. Immune system signaling was related to changes in pathways activated by inflammatory cytokines and alterations in signaling of cellular responses to stimuli, incoming from external molecular and physical signals. The top differentially up- and downregulated biological pathways and all the significantly deregulated pathways with DEGs involved are listed in **Supplementary Table 2**.

#### 3.4.2 A comparison of biological signaling pathways between metastatic and primary CRC tumors

“We wanted also to recognize which signaling pathways were altered in the metastatic CRC of patients with metastases in the liver compared to primary tumors. The top differentially up- and downregulated DEGs identified in Reactome v.83 are presented in **Table 5**.

**Table 5:** Top 10 up- and downregulated DEGs identified in mCRC compared to prim CRC; LogFC and corresponding p-values were estimated by the Reactome v83.

Identifier	Regulation	LogFC	p-value	Identifier	Regulation	LogFC	p-value
**CEBPB**	Up	1398.57	0.007	**TAP1**	Down	−2,113.43	<0.001
**RORA**	Up	528.98	0.007	**OAS1**	Down	−1,425.43	<0.001
**IFI30**	Up	5470.27	0.009	**TAPBP**	Down	−1,487.27	<0.001
**NFKBIA**	Up	7301.55	0.013	**ELK1**	Down	−324.75	<0.001
**LY96**	Up	516.91	0.016	**FADD**	Down	−454.94	<0.001
**MICA**	Up	405.78	0.020	**CCRL2**	Down	−324.96	<0.001
**MYD88**	Up	771.50	0.027	**SIGIRR**	Down	−718.52	<0.001
**C5**	Up	380.02	0.027	**PPARG**	Down	−2,518.31	<0.001
**HLA**−**DPA1**	Up	5721.58	0.037	**IRF2BP1**	Down	−164.00	<0.001
**IFITM3**	Up	21922.69	0.037	**PPIL2**	Down	−672.46	<0.001

The multi-omics analysis tool identified 88 differentially regulated biological pathways (**Supplementary Image 2**) related to signal transduction (ST) through cell surface receptors expressed on various cells in the CNS and the immune system triggering one or many cell responses from a single ligand binding. Disease alterations were associated with infectious diseases or aberrations in the signal transduction and immune system (IS) with its three main nodes. First is the innate IS through pathogen-associated molecular patterns (toll-like receptor (TLR) cascades) and induction of interferon-alpha/beta production. We also found changes in adaptive immune system in dysfunction of antigen processing and presentation and in cytokine signaling by interleukins (IL17). Other differently regulated pathways belong to developmental biology, gene expression, programmed cell death, cell cycle, and metabolism of carbohydrates, such as gluconeogenesis.

The connection between ST and IS signaling pathways was identified through signaling by receptor tyrosine kinases (RTKs), signaling by neurotrophic tropomyosin receptor kinases (NTRKs), and the following nuclear events (kinase and TF activation) and extracellular signal-regulated kinase (ERK)/mitogen activated protein kinase (MAPK) targets, MAPK activation, and IL17 signaling, as in the node for cytokine signaling in IS. Gene expression (generic transcription pathway and subsequent transcriptional activity of heterotrimer SMAD2/SMAD3:SAMD4) was also linked to ST by signaling of TGF-beta receptor complex. The top up- or downregulated biological pathways and all the significantly deregulated pathways with DEGs involved are listed in **Supplementary Table 3**.

#### 3.4.3 A comparison of signaling biological pathways between KRAS pathogenic mutation positive and negative CRC

The next aim of the study was to investigate the influence of KRAS pathogenic mutation on the CRC tissue DEGs (**Table 6**) and biological signaling pathways. KRAS-positive CRC tissues compared to negative counterparts were found to be involved in 46 biological signaling pathways (**Supplementary Image 3**), and all the significantly deregulated pathways with DEGs involved in are listed in **Supplementary Table 4**.

**Table 6:** Top 10 up- and downregulated DEGs identified in KRAS-positive compared to KRAS-negative CRC; logFC, and corresponding p-values were estimated by the Reactome v83.

Identifier	Regulation	LogFC	p-value	Identifier	Regulation	LogFC	p-value
**NR4A3**	Up	116.51	0.012	**IL12A**	Down	−26.90	0.009
**HAVCR2**	Up	44.47	0.014	**NMI**	Down	−666.91	0.043
**EGR2**	Up	68.78	0.014	**HSPD1**	Down	−49,615.67	0.057
**NFATC1**	Up	89.99	0.015	**GZMB**	Down	−1,050.27	0.087
**FOSL1**	Up	419.03	0.016	**MIF**	Down	−13,114.32	0.099
**SLC11A1**	Up	47.46	0.019	**EGF**	Down	−31.18	0.133
**IL10**	Up	34.80	0.024	**XCL1**	Down	−23.56	0.158
**ITGB2**	Up	191.13	0.025	**HLA-G**	Down	−587.70	0.183
**CLEC7A**	Up	94.42	0.027	**AIMP1**	Down	−731.05	0.196
**C5AR1**	Up	132.45	0.028	**CD40**	Down	−336.32	0.201

The main areas of altered pathways belong to disease and alterations in some infectious disease and signal transduction disease pathways. Signal transduction pathways frequently affected in malignant transformation (NOTCH, WNT, and mTORC1-mediated signaling) and signaling by RTKs, NTRKs, and subsequent nuclear events.

Biological pathways of the immune system were affected in all main nodes. The innate IS showed an inappropriate response in recognition of antimicrobial peptides and in ion influx/efflux at the host–pathogen interface. The adaptive IS was affected by signaling by B-cell receptor (BCR) and following downstream signaling events (as CD28 co-stimulation and CD28-dependent phosphoinositide 3-kinase (PI3K)/protein kinase B (AKT) signaling). The cytokine signaling was affected by DEGs that were found in signaling by CSF1 (M-CSF) in myeloid cells and/or signaling by interleukins, as IL12 signaling. Gene expression (transcription) was altered in RNA polymerase II transcription and generic transcription pathway and subsequent RUNX1-mediated regulation of genes. Furthermore, transcriptional regulation by the TFAP2 family of transcription factors and TP53-regulated transcription of several additional cell death genes.

Considering KRAS pathogenic mutation, signaling pathways influenced by few DEGs were found to belong to programmed cell death, developmental biology, transport of small molecules, and protein localization and mitochondrial protein import. The top up- or downregulated biological pathways are presented in **Supplementary Table 4**.

## 4 Discussion

Colorectal cancer development is multistage process that allows and adapts the changes/mutations occurring during cancer progression. The pathogenesis of CRC and its genetic instability is mediated through chromosomal instability, microsatellite instability, and epigenetic CIMP pathways (5). According to the CIMP state, three subclasses of genetic and epigenetic profiles have been identified, namely, CIMP-high (intense methylation of multiple genes, MSI, and BRAF mutations), CIMP-low (methylation of a limited group of genes and KRAS mutation), and CIMP-negative tissues (characterized with rare methylation and p53 mutation) (23). The regulation of several biological processes is disturbed, including cell proliferation, differentiation, angiogenesis, apoptosis and survival, and the responsible signaling pathways, such as EGFR/MAPK, Notch, PI3K, TGF-β, and Wnt signaling pathways, also do not exhibit their usual functions. Therefore, the exact identification of causal mutations and signaling pathways may trigger a novel preventive and therapeutic progress against CRC.

### 4.1 RNA-seq data evaluation and a brief analysis of signaling pathways responsible for CRC

The RNA-seq data were normalized, and 12 significant DEGs were selected as the most important in the cancer tissue when compared to the adjacent tissue. These DEGs are participating in cytokine–cytokine receptor interaction pathway that is a crucial aspect of inflammation and tumor immunology for CRC (24). Cytokines are released as a response to infection, inflammation, and/or immunity, as was demonstrated in consequential pathways (hsa-04061 and hsa04062). The intestinal epithelial cells represent an important defense line in protection against pathogens (25). The changes in intestinal immune network for IgA production (hsa04672) may influence the interaction of epithelial cells to pathogenic bacteria as *Helicobacter pylori* (hsa05120) and many others (hsa05163, hsa05134, hsa05171, hsa05131, hsa05133, hsa05135, hsa05132, and hsa05130), as was confirmed by our study.

The intestinal epithelial cells recognize the colonizing microbial community by TLRs that are very important in early innate immune defense mechanisms and trigger inflammatory pathways through intracellular signaling cascades, leading to the induction of genes encoding cytokines and chemokines, involved in antimicrobial host defense (26). Analogously, this RNA-seq DEGs analysis revealed that the significantly regulated DEGs were involved in TLR signaling pathway (hsa04620). The innate immune response to viral infections is mediated by the cytosolic DNA-sensing pathway (hsa04623) as is known as cGAS-STING pathway, and amendments in the pathway cascade drive inflammation-driven tumor growth (27) and participate at defense against viral infections (hsa05162, hsa05164, and hsa05167) and at virus-induced carcinogenesis (hsa05203). The changes in cytosolic DNA-sensing pathway (hsa04623) catalyzes the synthesis of cyclic dinucleotide cGAMP, which activates stimulator of IFN genes (STING) and mediates inflammation by inducing IFN1 production (IFN1) (22) and allowing innate immune response to infections, inflammation, and cancer (28). The cGAS and STING deficiency leads to eliminated or decreased level of IFN1 response to extrinsic cytosolic DNA, which may contribute to non-inflamed cancer microenvironment (28). Several colorectal human cell lines derived from adenocarcinoma have described defective or low STING pathway activity and poor stage of CRC (29).

Necroptosis (hsa04217) is another form of programmed cell death. Its mechanism is conformable to apoptosis and morphology analogous to necrosis. Necroptosis-related genes are strongly associated with TME of CRC, and patients carrying these gene changes may benefit from immunotherapy and have better prognosis of the disease (30).

The important parts of cancer research are TNF signaling pathway (hsa04668) and pathways in cancer (hsa05200). The TNF-α is a proinflammatory cytokine that often presents in the TME and is associated with chronic inflammation. Moreover, the proinflammatory NF-κB signaling is activated by the canonical pathway triggered by TNF-α, which results in the activation of p65 that regulates inflammatory responses (31). TNF-α promotes tumor angiogenesis and accelerates tumor metastasis, but the molecular mechanisms remain unclear (32). The second signaling pathway is associated with the MAPK and p53 signaling pathways (33).

We also identified lipid and atherosclerosis (hsa05417) pathway, in which lipids are energy reservoirs that can control homeostasis, transcriptional, and enzymatic networks, and inflammatory response and reprogramming of the lipid metabolism are two hallmarks of cancer (34). Atherosclerosis is similar to solid tumors because of the content of macrophages that participate in acidosis, anaerobic metabolism, and inflammatory process (35). The pathogenesis of rheumatoid arthritis (hsa05323) is also linked with inflammation and presence of survivin that inhibits apoptosis and contributes to persistence of autoreactive T cells and the tumor-like phenotype of fibroblast-like synoviocytes. The overexpression of survivin also affect signaling pathways, such as STAT3 and PIK3/Akt, and is involved in the severity of rheumatoid arthritis (36).

### 4.2 RNA-seq data verification on larger cohort of CRC patients

Six genes were selected for validation of RNA-seq data, such as IL6R, BLNK, TNFSF10, CCR4, MICB, and RIPK2. There was a correlation between RNA-seq and validation cohort for the IL6R, BLNK, and TNFSF10 DEGs when cancer and control tissues were compared. These three DEGs were also identified as top 12 DEGs. Other three DEGs had a weak correlation between the tested methods. All six DEGs had also a weak correlation between the metastatic and primary tissues and between KRAS-positive versus KRAS-negative cancers.

0This study analyzed IL6R expression on a large cohort of CRC patients; we found a significantly lower expression in the cancer group and a non-significantly higher expression in the metastatic group compared to primary cancer and a slightly lower expression in the KRAS-positive group. IL6R is expressed only in cells as hepatocytes and certain leukocytes, whereas its ligand IL6 is expressed in a wide variety of cell types. IL6R transduction pathway is mediated through activated STAT3, MAPK, and PI3K activation (37). Authors found that colorectal cancer cell lines express IL6 and IL6R and co-receptor gp130 (38), and higher levels of IL6R mRNA expression were found in HER2-positive breast cancer lines and in immortalized cells derived from nasopharyngeal epithelium with activated STAT3 (39). This study revealed significantly lower IL6R and lower STAT3 expression.

B-cell linker protein (BLNK) is crucial for B-cell receptor signaling pathway and mediates B-cell apoptosis (40). BLNK overexpression was found in approximately 70% of CRC tissues where its oncogenic activity via RAS/ERK pathway has been reported (41). BLNK overexpression was an independent risk factor for CRC recurrence (42). A reduced expression of BLNK was found in increased migration and invasion of CRC cells (43). BLNK expression on a large cohort of CRC patients was found to be significantly lower in the cancer group and equal expression in mCRC when compared to primary cancer and a higher expression in the KRAS-positive group.

Tumor necrosis factor (TNF) superfamily member 10 (TNFSF10), known as tumor necrosis factor (TNF)-related apoptosis-inducing ligand (TRAIL) or ApoEL, is able to induce cell apoptosis in various types of tumor cells (44) by receptor oligomerization and recruitment of the FADD and caspase 8 and 10 (45). TNFSF10 expression on a large cohort of CRC patients was found to be significantly lower in the cancer group compared to the control group and non-significantly lower in metastatic and KRAS-positive group. Downregulated expression of TNFSF10 was found also in transcriptomic study of CRC compared to normal data available in TCGA database (46).

MICB or MHC class I chain-related B molecule is one of the ligands of NKG2D receptor that exists in NK cells and CD8^+^ T cells. MICB is expressed by the intestinal epithelium or epithelial tumors, and their role is in the immunosurveillance and mediates antitumor response (47). MICB expression on a large cohort of CRC patients uncovered its significantly lower expression in the cancer group compared to the control group, non-significantly lower expression in the metastatic group, and weakly significantly lower expression in the KRAS-positive group. A study of MICB expression had shown a significantly high expression that was associated with tumor size and better overall survival (48). Another study revealed worse survival of patients with CRC with downregulated MICB because the tumor evades recognition by the immune system (49).

Another molecule used for the validation of RNA-seq data was the receptor-interacting serine/threonine-protein kinase-2 (RIPK2). RIPK was found to be involved in solid tumors and have a role in different pathways of immune and inflammatory responses (50). The RIPK2 expression on a large cohort of CRC patients was found significantly increased in the cancer group, non-significantly higher expression in the metastatic group, and non-significantly lower expression in the KRAS-positive group. High expression of RIPK was associated with high expression of VEGFA and increased mortality and has a potential in targeted therapy (51).

The role of CC chemokine receptor (CCcR4) was identified in normal and tumor immunity and belongs to the G-protein-coupled receptor family. The binding of chemokine ligands trigger its function in human autoimmune diseases, such as atopic dermatitis, asthma, and cutaneous T-cell lymphoma (CTCL), and is expected to be a novel therapeutic target for cancer immunotherapy (52). We identified a non-significant expression of CCR4 in each group; CCR4 expression was equal in the cancer group when compared to the control group, slightly higher in the metastatic group, and lower in the KRAS-positive group.

### 4.3 Reactome data analysis identification of DEGs in CRC compared to adjacent tissue

We further aimed to investigate which changes in signaling pathways could be responsible for the development of CRC. Here, we discuss an altered gene expression and signaling pathways that were significantly changed in the CRC tissue compared to the control adjacent tissue. Using *in silico* Reactome analysis, we identified the significantly upregulated genes, namely, CDK2, MYC, GPI, CD44, NOD1, UBE2N, PRKRA, and IRAK2, and the significantly downregulated gene, IRF7 gene (**Supplementary Table 2**).

Cyclin-dependent kinase 2 (CDK2) is a DNA damage signaling kinase, which phosphorylates proteins in many cellular processes and is hyperactivated in most cancers (53). UBE2N interacts with BRCA1, and its expression serves as a potential biomarker of response to poly(ADP-ribose) polymerase (PARP) inhibitors and other DNA-repair-targeted therapies in breast cancer (54).

Cell cycle signaling pathways importantly participate in cell cycle division. Most cancer cells have defects in G1 and G2 checkpoints, and DNA damage triggers the ATM/CHK2/p53 pathway. The weakening of G2 checkpoint leads to chromosomal instability, and dysregulated cell-cycle-related genes can be a reason for uncontrolled cell growth and proliferation that are a signs of cancer cells (55).

The *in silico* analysis of cancer cells found changes in biogenesis in miRNA and siRNA, and mainly TP53 gene expression was disturbed. The TP53 is a key tumor suppressor that regulates different cellular responses to protect against cancer development. Colorectal cancer is reported with 43% of mutations in TP53 gene, and mostly missense mutations impair wild-type p53 function (loss-of-function). Invasive and metastatic cancers may provide gain-of-function activities with more aggressive phenotype acquired by clonal evolution of cancer stem cell (56). Patients with breast cancer carrying the TP53 mutation were diagnosed with a higher glucose-6-phosphate isomerase (GPI) gene expression that indicates higher level of glycolysis in tumor cells and correlates with the degree of tumor malignancy (57). Furthermore, MYC overexpression was observed in up to 70%–80% of CRC (58). Numerous studies sustained a chemoresistance in tumor cells expressing high levels of MYC and activation of WNT signaling pathway. Gaggianesi et al. demonstrated that dual indirect targeting of CD44 and MYC in CRC stem cells, using PI3K and CDK inhibitors, reduces the survival and clonogenic activity of cancer stem cells, regardless of the mutational background (59). Similarly, MIF overexpression leads to excessive signaling through CD74 surface receptor and formation of complex with CD44 that initiates the ERK/MAPK signaling pathway. Chemokine-like function of MIF consists of the recruitment of immune cells and mediation of acute and chronic inflammatory diseases, and tumor progression and development. CRC patients with MIF overexpression in lymph nodes had a shorter survival time after surgery (60).

PRKRA is a core component in the miRNA/siRNA biogenesis and is known as a cellular protein activator of PKR kinase (also known as EIF2AK2) in a dsRNA-independent manner in response to cellular stress. PKR kinase can then induce the expression of type I interferons (IFNs). Increased expression of PRKRA can be associated with worse survival of colorectal cancer patients (61).

Pathways associated with signal transduction were linked to the activation of TNFSF10 (alias TRAIL) and initiation of apoptosis through FADD domain complex and effector caspases (casp-3, casp-6, and casp-7) (62). TNFSF10 can also induce non-apoptotic signaling through the activation of proinflammatory pathways, including NF-KB, PI3K/Akt, and MAPK such as JNK, ERK, and p38 (63).

Intestinal epithelial cells and immune cells recognize exogenous and endogenous stimuli through TLRs. IRAK2 is a key regulator of interleukin-1 receptor (IL1R)/TLR-mediated inflammation and is involved in NF-κB and MAPK signaling pathways (64). In addition to TLRs, other pattern recognition molecules (PRRs) (65) are often present in cells. This study revealed overexpressed DEGs such as UBE2N, RIPK2, NOD1, and IRAK2, which points to the altered expression of the other two PRRs. NOD1 and NOD2 are bacterial sensors that trigger proinflammatory signaling mediated by receptor-interacting protein kinase 2 (RIPK2). UBE2N is a ubiquitin enzyme that eases the production of non-embedded polyubiquitin chains and serves as an activator for RIG-I on virus infection (66). The recognition of pathogenic microorganisms by PRRs induces the activation and translocation of IRF7 (interferon regulatory factor 7) to nuclear, leading to IFN-I secretion. IRF7 has opposite functions in carcinogenesis (67). This study indicates a significant downregulation of IRF7 in CRC. Otherwise, the downregulation of IRF7 expression promoted polarization of tumor-associated macrophages (TAMs), which produce anti-inflammatory factors to enhance breast cancer development by promoting immune escape, proliferation, and migration of cancer cells (68).

### 4.4 Reactome data analysis identification of DEGs in metastatic CRC tissue compared to primary tumor

Another part of the study was a comparison of signaling pathways in metastatic versus primary CRC. The Reactome analysis uncovered the following pathways that we discussed in a broader context of the disease. Nevertheless, the study results did not influence the treatment of patients with mCRC; patients were administrated with standard therapy prescribed by the experienced oncologist.

Using *in silico* Reactome analysis, we characterized DEGs in tumors that were able to invade the liver and form a metastasis. We found a significant downregulation of genes, such as CASP8, FADD, ELK1, ERBB2, IRAK1, SIGIRR, TAP1, TAP2, TAPBP, HLA-E, STAT3, MYC, IFNAR1, PPARG, PPIL2, and IL17RE (**Supplementary Table 3**).

The downregulation of FADD and CASP8 might bring the evidence of blocked apoptosis in mCRC in programmed cell death signaling that was discussed above.

Signal transduction (ST) pathways were altered in ERK/MAPK pathways where we found significantly decreased expression of ELK1 in mCRC. ELK1 is usually upregulated in cancer. Nevertheless, ELK1 expression almost disappeared in the middle stage of G1 phase and at the end of S phase (69). The analysis also uncovered a reduced expression of ERBB2 (HER2) gene that plays an essential role in the regulation of cellular proliferation, differentiation, and migration via the same signaling pathways, such as ERK/MAPK and PI3K/Akt/mTOR (70). It is hypothesized that HER2 expression is abrogated during EMT by chromatin-based epigenetic silencing of ERBB2 gene. Subsequently, tumors become resistant to HER2-targeted therapies (71). In line with previous findings, a significant downregulation of STAT3 was found in oncogenic MAPK signaling represented by RAS/RAF/MAPK cascade that is important in regulating cellular proliferation, differentiation, and survival by MAP2K mutants, such as BRAF, RAF1, and RAS gene alterations (72). Other pathways were likely responsible for the metastatic progression of CRC in the cohort, so STAT3 molecules or inhibitors would not be applicable here. Controversially, a reduced MYC expression has been found in mCRC in comparison to primary CRC. Reduced MYC expression was found in cancer cells localized in the area distant from blood vessels where TME contains limited levels of oxygen and glucose. This might be a strategy of cancer cells to survive under conditions of limited energy sources (73).

The most signaling pathways detected in this part of *in silico* analysis were associated with immune system (IS). Innate IS response in CRC tissues have increased expression of TLRs because of the presence of chronic inflammation, microbial pathogens-induced changes in metabolism, TME, and genotoxic response (74). Commensal microflora in intestinal homeostasis can modulate TLR signaling through single immunoglobulin IL-1 receptor-related molecule (SIGIRR) that is a negative regulator for TLR-IL1R signaling. Hyperactivation of TLR-IL1R-mediated Akt-mTOR signaling in SIGIRR-deficient tumors leads to cell cycle progression, loss of heterozygosity of APC, and tumor initiation (75).

TLR-IL1R signaling is also important in the coordination of the early immune response to pathogens that is mediated by the protein myeloid differentiation primary response protein 88 (MyD88) where IL-1R-associated kinase (IRAK) proteins are employed (76). Catalytically activated IRAK1 and IRAK4 with the participation of MyD88 interact with TNF-receptor-associated factor 6 (TRAF6) and drive NF-kB and MAPK pathways that results in the production of proinflammatory cytokines (77). IRAK1 has been upregulated in many cancers and is considered as one possible target in cancer therapy. However, low expression levels of IRAK1 may cause the failure of targeted cancer therapy.

We also identified changes in cytokine signaling, especially the downregulation of interleukin 17 (IL-17) receptor E (IL17RE). IL-17RE is expressed in T_H_17 cells and in the epithelial cells themselves, and its ligand is interleukin 17C (IL-17C). IL17C secretion maintains an autocrine loop in the epithelium, thereby enhancing innate immune barriers (78). A reduction or attenuation in IL-17R induces MAPK signaling pathway in the downregulation of ERK2 expression and downstream targets (ELK1, ETS2, RSK, MNK, and PLA2). Tumors with IL-17R deletion express molecular markers for tumor growth, invasion, and metastasis (79), which was also confirmed by our study.

We also identified a decreased expression of IFNAR1 chain of the IFN1 receptor that is activated by JAK-STAT and other signaling pathways. Downregulation of IFNAR1 is often present in the malignant cells and in TME, where IFN1 pathway is suppressed (80).

Adaptive immune response was altered in class I MHC-mediated antigen processing and presentation,. We identified downregulation of MHC class I, which is frequent in tumors and might be an intrinsic mechanism of acquired resistance to immunotherapy (81, 82). Subsequently, the downregulation of transporters associated with antigen processing (TAP1/TAP2 and TAPBP) is also logical, which was identified in the study. We also identified a downregulation of HLA-E expression. Studies showed that the loss of MHC class I expression is accompanied with the loss of HLA-E or HLA-G expression, mean significantly better overall, and disease-free survival for the patients (83).

Signaling pathways in gene expression were associated with transcription of several transcription factors, such as peroxisome proliferator-activated receptors (PPARs) that act as antagonists in transcription of immunity and inflammation factors (84). We identified significantly low expression of PPARG in mCRC. Authors found a lack of PPARG expression in 30% of primary CRCs that strongly correlates with promoter methylation and was found in patients with poor prognosis (85). In contrast, other authors found no difference in disease progression or survival; therefore, PPARG is not an active agent for the treatment of metastatic colorectal cancer (84). We also identified a peptidylprolyl isomerase (cyclophilin)-like 2 (PPIL2) molecule that was downregulated in mRNA biogenesis and metabolism. PPIL2 is an ubiquitin ligase and probably participates in breast cancer metastasis in animal models because its downregulation led to increased migration of human breast cancer cells (86).

The interesting revelation in mCRC was the connection between ST and IS signaling pathways mediated by receptor tyrosine kinases signaling, signaling by NTRKs and following nuclear events (kinase and TF activation) and ERK/MAPK targets, MAP kinase activation, and IL17 signaling, as in the node for cytokine signaling in IS. This molecular interaction seems to be a hot candidate in the altered molecular profile of basic tumor, which derived distant metastases when compared to primary CRC. Changes in signaling pathways can allow individual cells to detach from the original tumor and spread to other parts of the body.

### 4.5 Reactome data analysis identification of DEGs in KRAS-positive versus KRAS-negative CRC tissue

The data analysis of KRAS-positive versus KRAS-negative CRC tissues has not revealed any significantly up- or downregulated DEGs. Therefore, we present the top up- and downregulated pathways according to FDR below 0.05 (**Supplementary Table 4**).

KRAS gene is highly mutated in up to 50% of colorectal cancer. The activation of KRAS proteins can be performed by growth factors, receptor tyrosine kinase (RTK), chemokines, and Ca^2+^ ions. The downstream signaling pathways of activated KRAS protein are the RAF-MEK-ERK (MAPK) signaling pathway, PI3K-Akt-mTOR signaling pathway, and other signaling pathways (87). KRAS mutation has a significant influence on the progression and treatment of colorectal cancer (88). KRAS-positive tumors had alterations in two main categories of the disease signaling pathways. Upregulation in pathways is an anti-inflammatory response favoring *Leishmania* parasite infection and mitigation of host antiviral defense response. These pathways can point that T-cell response and antigen presentation were reduced in KRAS-positive tissues, as described by Liu et al. (89).

The alterations in signal transduction pathways confirmed the role of KRAS in signaling by Notch, Wnt, mTOR, and NTRKs that function as a network in CRC stem cells (90). The major downstream effectors in KRAS oncogenic signaling is the TGF-β pathway that has been identified as the top pathway enriched in invasive and metastatic tumors (91).

The Reactome analysis identified a wide involvement of the immune system in cancer tissues carrying KRAS mutations. TME is present with inflammation, high levels of inflammatory cytokines and chemokines, and is infiltrated with multiple immune cells (92). We identified upregulation in C-type lectin receptor (CLR) signaling, as dectin-1 (CLEC7A) that increases production of cytokines and chemokines through activation of NF‐κB via caspases and MAPK, and nuclear factor of activated T cells (NFAT) pathways and induces reactive oxygen species (ROS) production. The detailed process is described in the review (93). Dectin-1 expression in TAMs promoted their suppression of anti-tumor immunity and toleration of tumor cells in pancreatic ductal adenocarcinoma, making Dectin-1 an interesting target for immunotherapy (94).

Adaptive immunity, with its components B- and T-cells, is involved in pathogen clearing. T-cells are activated by antigen recognition by T-cell receptor and by costimulatory molecule, such as CD28 receptors, and may activate PI3K/Akt signaling that promotes cytokine transcription, survival, cell-cycle entry, and growth (95). Signaling by B-cell receptor (BCR) and downstream phosphorylation of ITAMs lead to NFAT activation by calcineurin, IP3, and/or PIP3, and NF-κB is activated via PKC, Ras is activated via RasGRP, and Akt is activated via PDK1 (95). Cytokine signaling regulates and mediates immunity, inflammation, and hematopoiesis. Reactome analysis also identified changes in signaling by CSF1 (macrophage colony stimulating factor, M-CSF) in myeloid cells and/or signaling by interleukins, such as IL12 signaling. Tumors frequently contain TAMs, which contribute to their development and progression and are useful in antitumoral therapy (96).

DEGs enriched in signaling pathways of gene expression (transcription) revealed RUNX1-mediated regulation of cell differentiation genes, including keratinocytes, myeloid and megakaryotic progenitors, and regulatory T and B lymphocytes (97).

The roles of transcription factors with RUNX in EMT and tumor progression should be more studied (98, 99). Transcriptional regulation by the TFAP2 family of transcription factors was found to be reduced in high-grade colorectal adenocarcinomas (100). Next, we found alterations in TP53 regulation of transcription of cell death genes with uncertain role in apoptosis. Authors found that patients with a high expression of TP53 in KRAS-positive CRC had a poor prognosis of the disease (101). An upregulation in the FOXO-mediated transcription of cell death genes was also identified in the study. FOXO transcription factors participate in cell proliferation and in cell apoptosis including the control of autophagy, metabolism, inflammation, and differentiation (102).

The alterations in transport of small molecules through transporters, such as ATP-powered pumps, ion channels, and transporters, represent the imbalance in the transport of bile salts, organic acids, metal ions, and amine compounds, and subsequent organic cation transport and/or organic cation/anion/zwitterion transport, and metal ion SLC transporters, and protein localization and mitochondrial protein import. It was proven that KRAS mutations in CRC cells correlate with the higher amino acid uptake when compared to KRAS wild-type colorectal cells (103).

## 5 Conclusion

The understanding of pathology and altered gene expression of CRC would help to identify the key point in treatment selection, monitoring, and prevention of disease relapse. Our data may partially contribute to clarifying the function of the immune system in the TME of CRC.

The difference between CRC and control adjacent tissue identified the main altered pathways in CRC. A significant participation of tumor-associated macrophages was seen in TME. The initiation of the inflammatory response likely plays the role in altered gene expression of TP53, and the accumulation of other gene mutations probably affects subsequent errors in DNA repair and cell cycle pathways. The *in silico* analysis revealed the changes at the level of surface receptors of epithelial or immune cells through which they interact with the colonizing microbiome. The subsequent reaction of macrophages supports the immune response and inflammation; the permanent production of proinflammatory cytokines and chemokines leads to further changes in cellular pathways. The emergence of mutations and the selection of tumor clones with blocked apoptosis allow mistakes in the cell cycle and accumulation of mutations and uncontrolled proliferation of tumor cells.

Analysis of metastatic tumor tissues compared to the primary tumor showed persistent changes in the expression of cellular surface receptors, chronic inflammation, and the presence of proinflammatory cytokines (e.g., IL-17) and changes in downstream signaling pathways (TGF-β and MAPK signaling). The main finding was the influence of the immune system to signal transduction pathways that may be linked to the activity of tumor-associated macrophages. Unfortunately, this study does not analyze secondary metastatic tumors. Therefore, the study does not reflect the clonal evolution and associated signaling pathways that lead to detachment of specific tumor cell. We indicate only basic molecular pathways that enabled the formation of metastatic cancer stem cells.

KRAS mutations also affected signal transduction pathways, particularly Notch1 and Wnt signaling pathways, the innate and adaptive immune system, and cytokine production. The gene expression level was mainly affected by RUNX1-mediated regulation of cellular differentiation and by regulation of TP53 transcription.

A precise identification of signaling pathways responsible for CRC may help in the selection of personalized pharmacological treatment because many targeted therapies to specific cancers have been developed.

## Data availability statement

The original contributions presented in the study are publicly available. This data can be found here: https://data.mendeley.com/datasets/xjxkyzm6vr/1.

## Ethics statement

The studies involving humans were approved by Ethics committee in the Jessenius Faculty of Medicine in Martin, Comenius University in Bratislava, number 1863/2016. The studies were conducted in accordance with the local legislation and institutional requirements. The participants provided their written informed consent to participate in this study.

## Author contributions

VH performed signaling pathways analysis and wrote the manuscript; DL performed RNAseq experiments; MG performed bioinformatic analyses and reviewed the manuscript; ZK and VH performed qPCR experiments; PM, JMi, and EK collected patient´s samples; ET assisted the experiments; MK and JMa performed histopathological evaluation; LL reviewed and revised the manuscript; ZL designed the study, ensured the financial funding, supervised the data analysis and reviewed the manuscript. All authors contributed to the article and approved the submitted version.
