# Supplementary material for: Differential gene expression of immunity and inflammation genes in colorectal cancer using targeted RNA sequencing
Source: Front Oncol. 2023 Oct 5;13:1206482. doi: 10.3389/fonc.2023.1206482 (PMC10586664; doi:10.3389/fonc.2023.1206482)
Supplement: Supplementary file 1 [file DataSheet_1.pdf]

report_coca_FC__March15_2023.R

mg

2023-03-15

indexplot, avHK

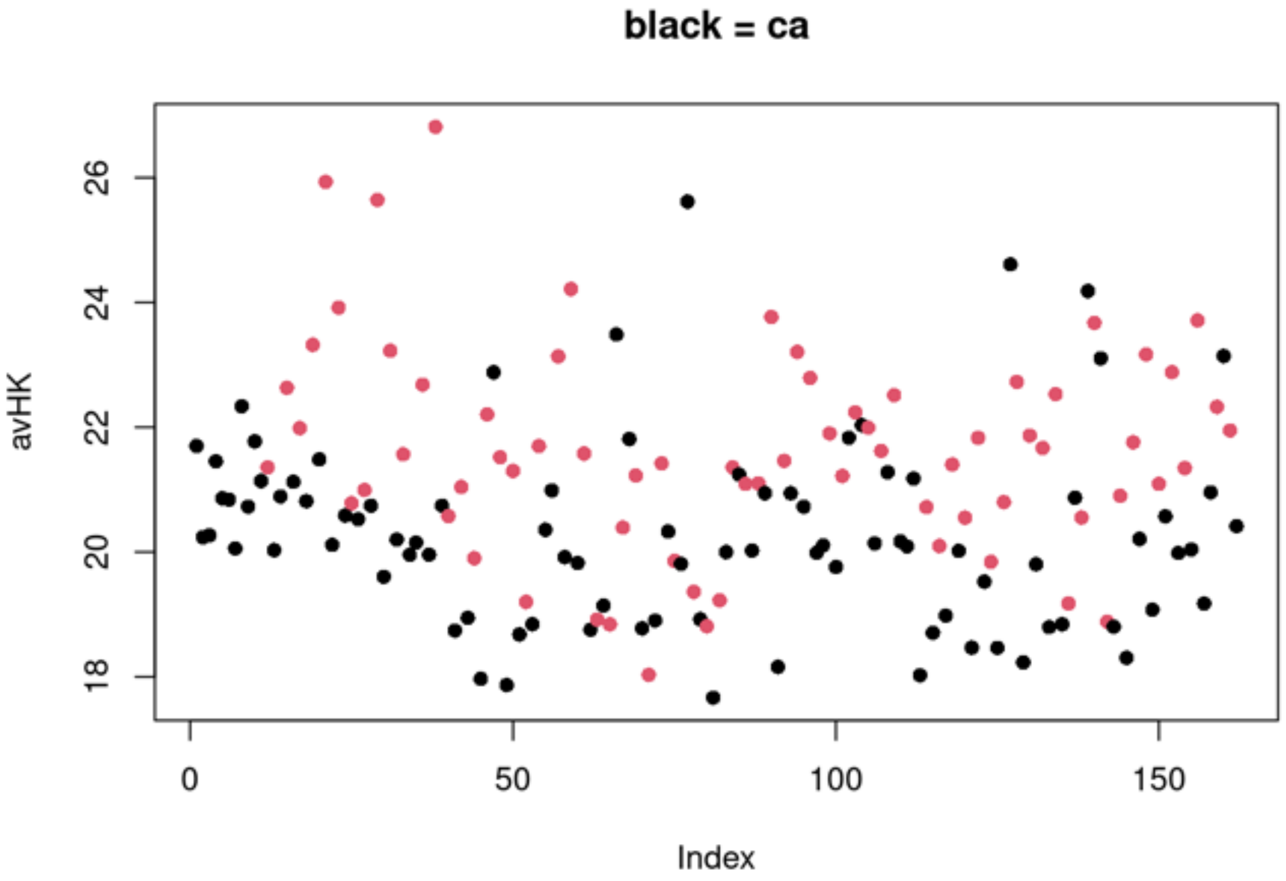

ca vs co

EDA of logFC

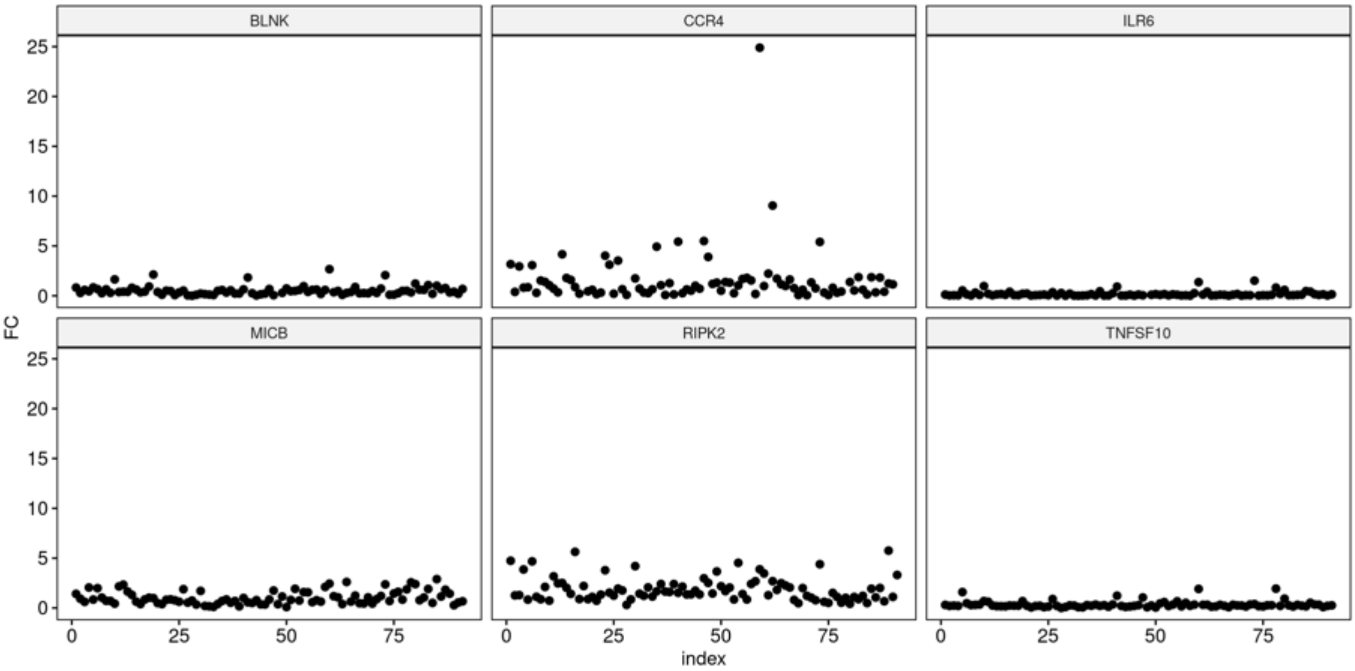
BLNK
CCR4
ILR6
MICB
RIPK2
TNFSF10
FC
index
0
5
10
15
20
25
50
75

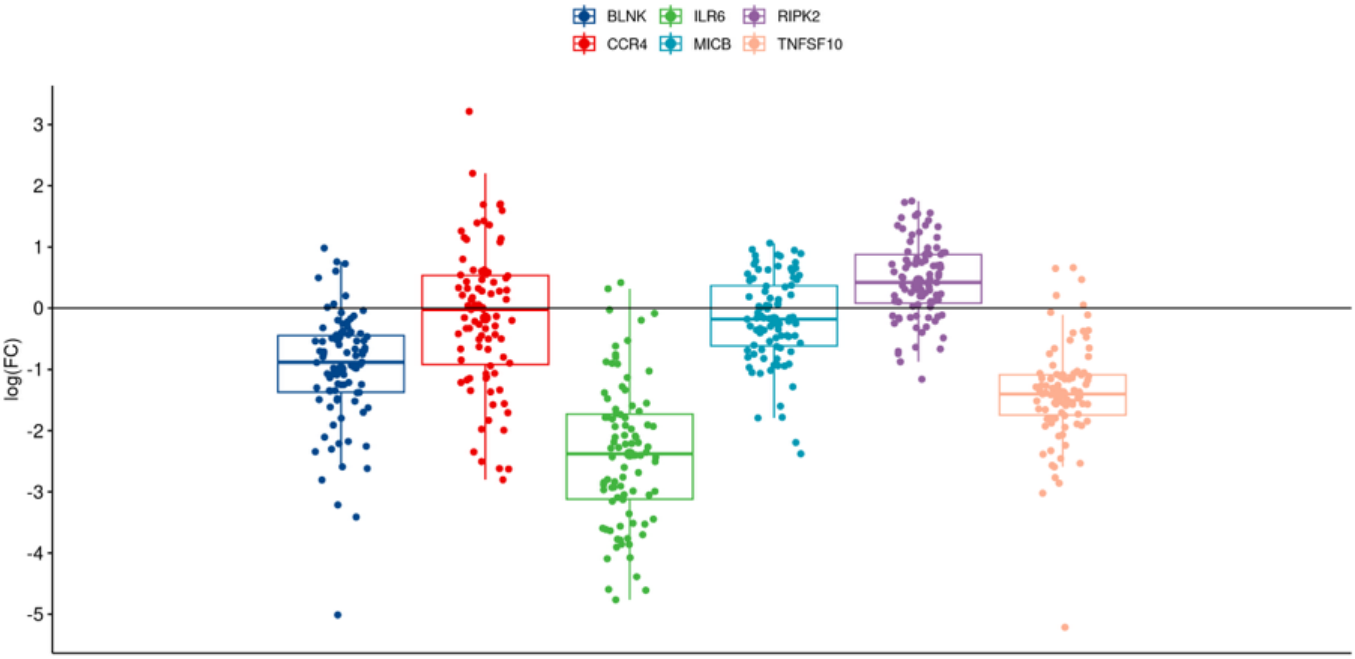
BLNK
ILR6
RIPK2
CCR4
MICB
TNFSF10
log(FC)
3
2
1
0
-1
-2
-3
-4
-5

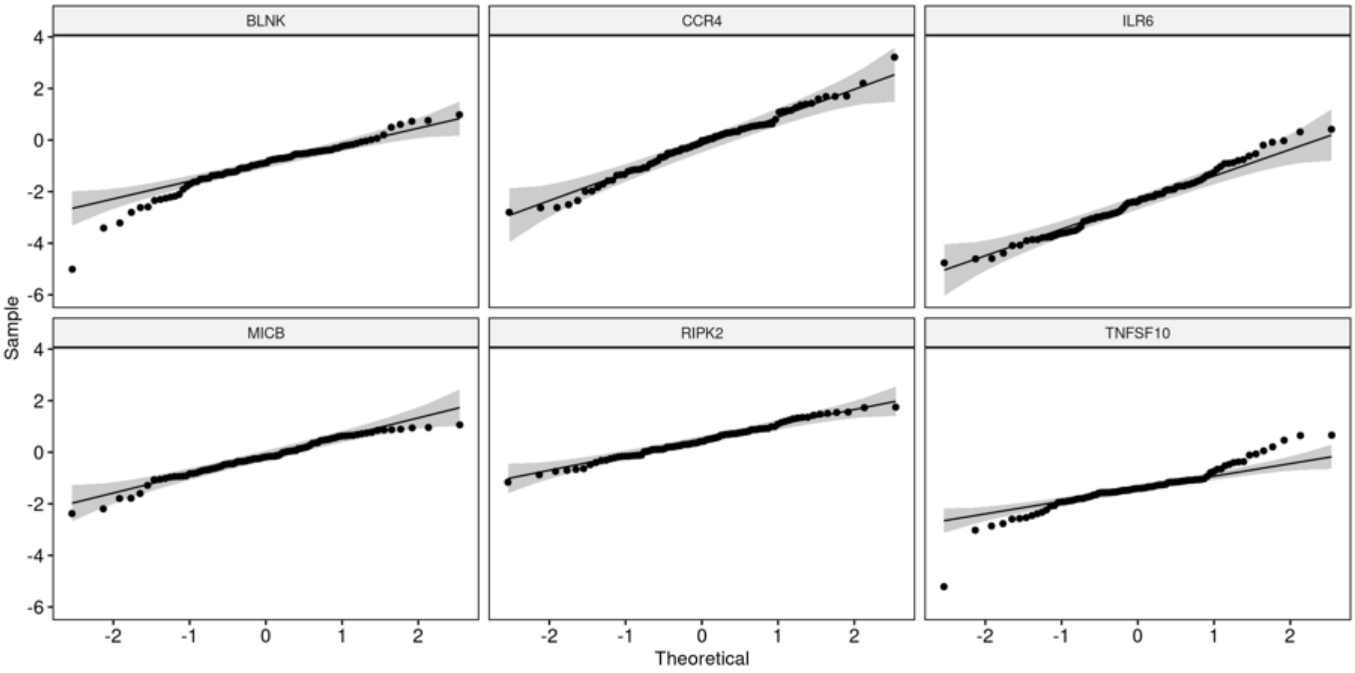
BLNK
CCR4
ILR6
MICB
RIPK2
TNFSF10
Sample
Theoretical
4
2
0
-2
-4
-6
-1
1

Descriptive stat of log(FC)

Characteristic	N = 91[1]
BLNK	-0.88 (-1.37, -0.45)
Unknown	1
CCR4	-0.02 (-0.92, 0.54)
Unknown	4
ILR6	-2.38 (-3.12, -1.73)
Unknown	1
MICB	-0.18 (-0.61, 0.37)
RIPK2	0.42 (0.08, 0.88)
TNFSF10	-1.40 (-1.74, -1.09)

[1]Median (IQR)

Wilcoxon test; H0: mean log(FC) = 0

gene	p.value
BLNK	0.0000000000000920252470
CCR4	0.2776524937021490613276
ILR6	0.0000000000000002398323
MICB	0.0437421753748651678806
RIPK2	0.0000000110847009135223
TNFSF10	0.0000000000000004239486

mts vs primary

EDA of logFC

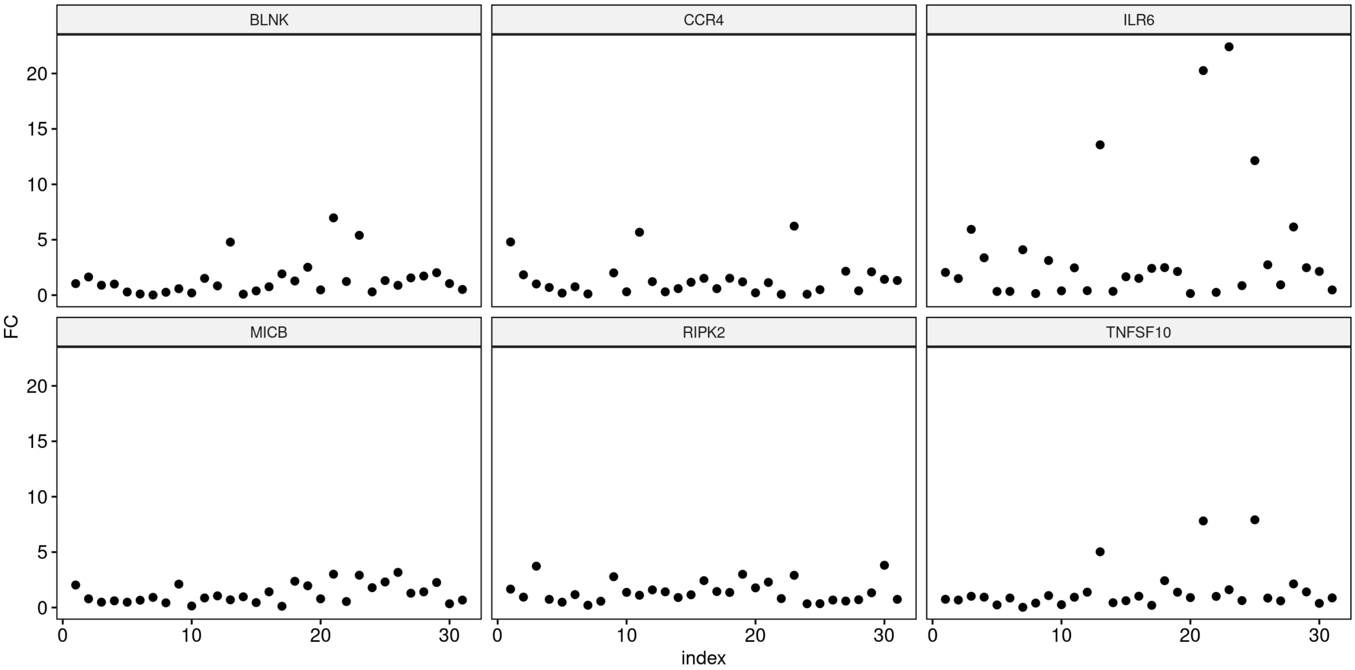
BLNK
CCR4
ILR6
MICB
RIPK2
TNFSF10
FC
index

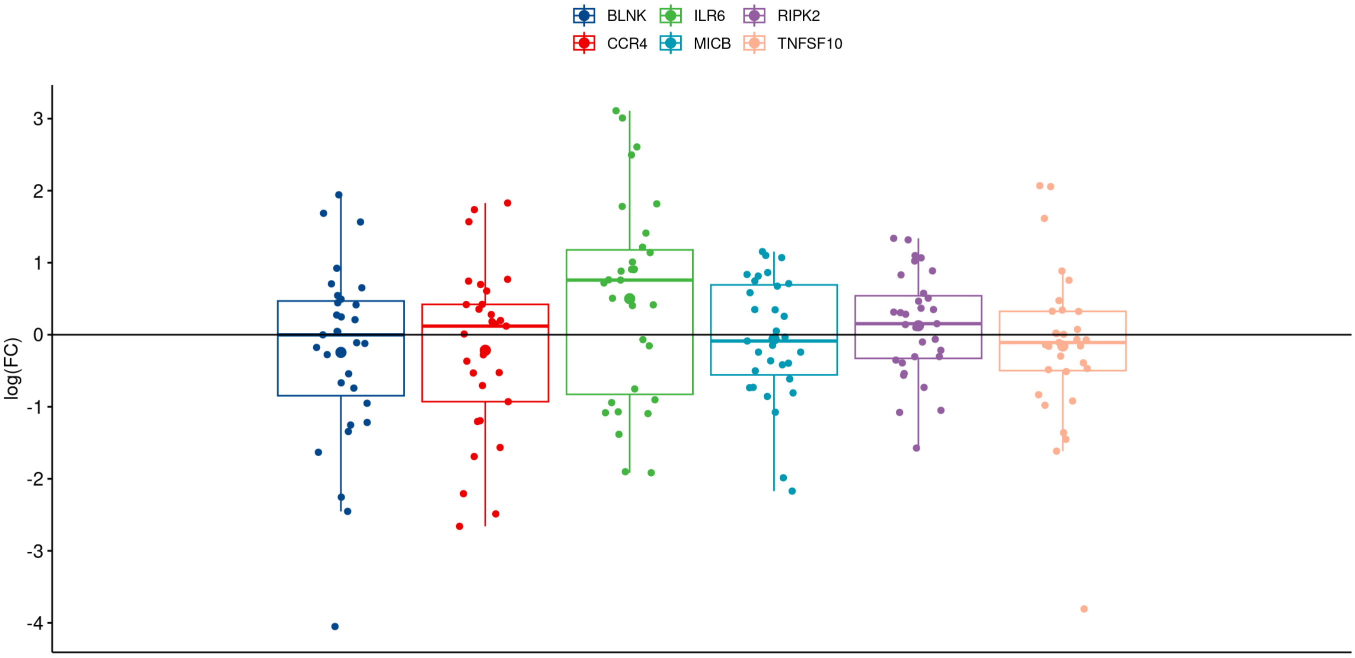
BLNK
ILR6
RIPK2
CCR4
MICB
TNFSF10
log(FC)

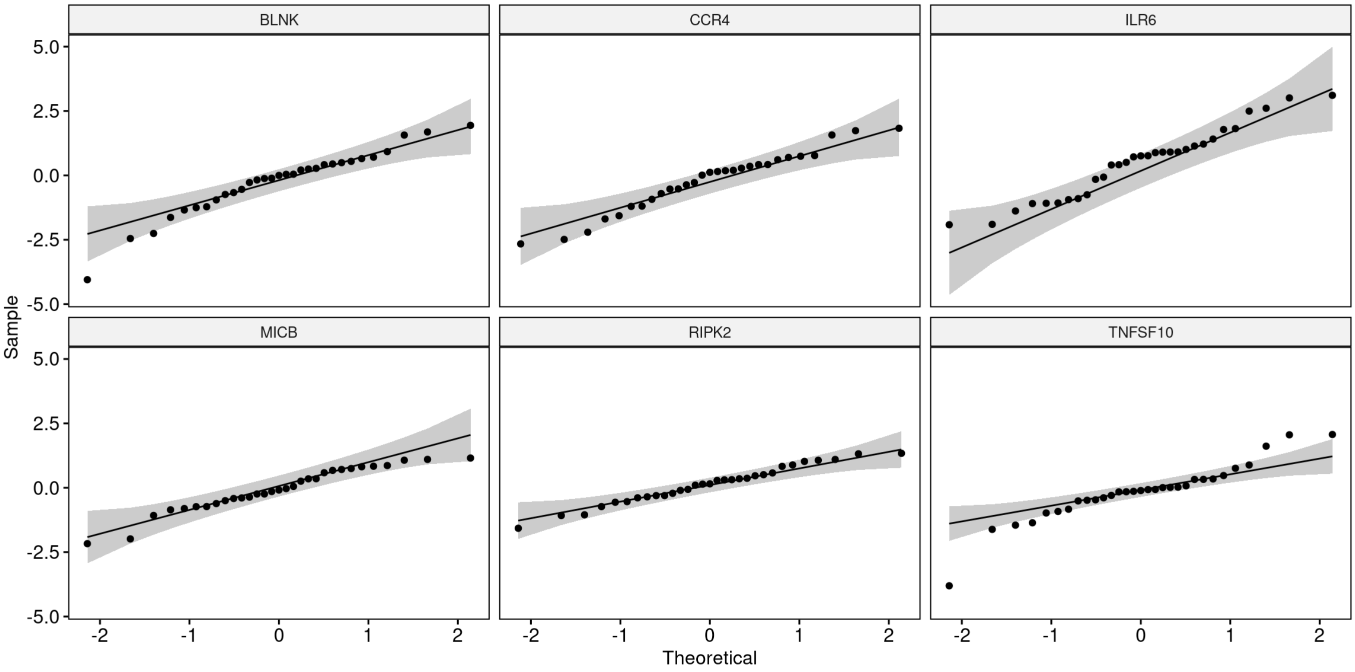
BLNK
CCR4
ILR6
MICB
RIPK2
TNFSF10
Sample
Theoretical

Descriptive stat of log(FC)

Characteristic	N = 31[1]
BLNK	0.00 (-0.85, 0.47)
CCR4	0.12 (-0.93, 0.42)
Unknown	2
ILR6	0.76 (-0.83, 1.18)
MICB	-0.09 (-0.56, 0.69)
RIPK2	0.15 (-0.33, 0.54)
TNFSF10	-0.11 (-0.50, 0.32)

[1]Median (IQR)

Wilcoxon test; H0: mean log(FC) = 0

gene	p.value
BLNK	0.5167645
CCR4	0.5503523
ILR6	0.1066251
MICB	0.9768866
RIPK2	0.3175717
TNFSF10	0.3175717

KRAS+ vs KRAS-

EDA of logFC

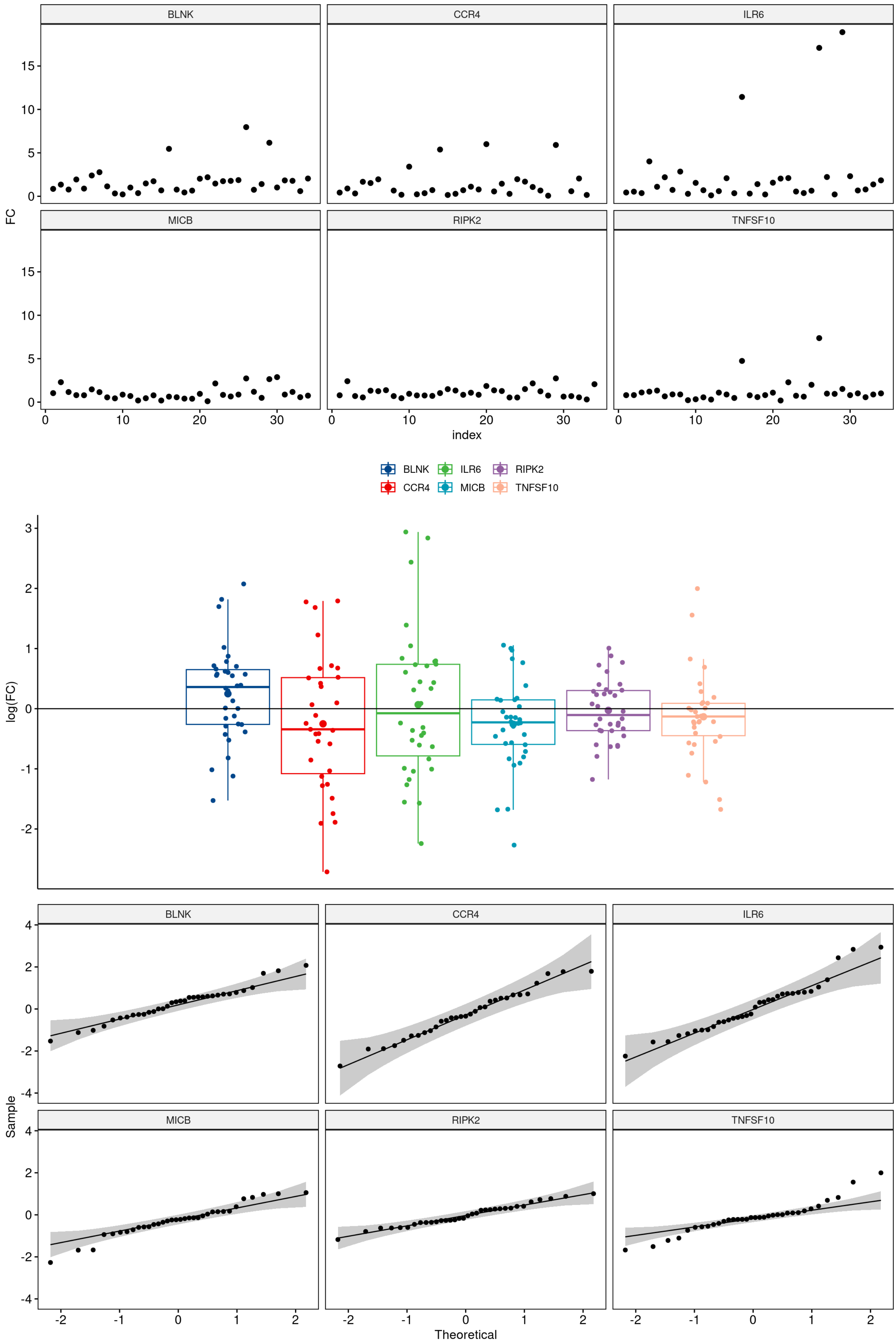

BLNK
CCR4
ILR6
MICB
RIPK2
TNFSF10
FC
index
log(FC)
Sample
Theoretical

Descriptive stat of log(FC)

Characteristic	N = 34[1]
BLNK	0.36 (-0.26, 0.65)
CCR4	-0.34 (-1.08, 0.52)
Unknown	3
ILR6	-0.08 (-0.78, 0.74)
MICB	-0.23 (-0.59, 0.15)
RIPK2	-0.10 (-0.36, 0.30)
TNFSF10	-0.13 (-0.45, 0.09)

[1]Median (IQR)

Wilcoxon test; H0: mean log(FC) = 0

gene	p.value
BLNK	0.05476904
CCR4	0.28106469
ILR6	1.00000000
MICB	0.05043983
RIPK2	0.72295671
TNFSF10	0.07234450
