# Supplementary material for: Differential gene expression of immunity and inflammation genes in colorectal cancer using targeted RNA sequencing
Source: Front Oncol. 2023 Oct 5;13:1206482. doi: 10.3389/fonc.2023.1206482 (PMC10586664; doi:10.3389/fonc.2023.1206482)
Supplement: Supplementary file 2 [file DataSheet_2.pdf]

report_coca_rtPCR_vs_RNAseq__March15_2023.R

mg

2023-03-15

ca vs co

crossplot of FC

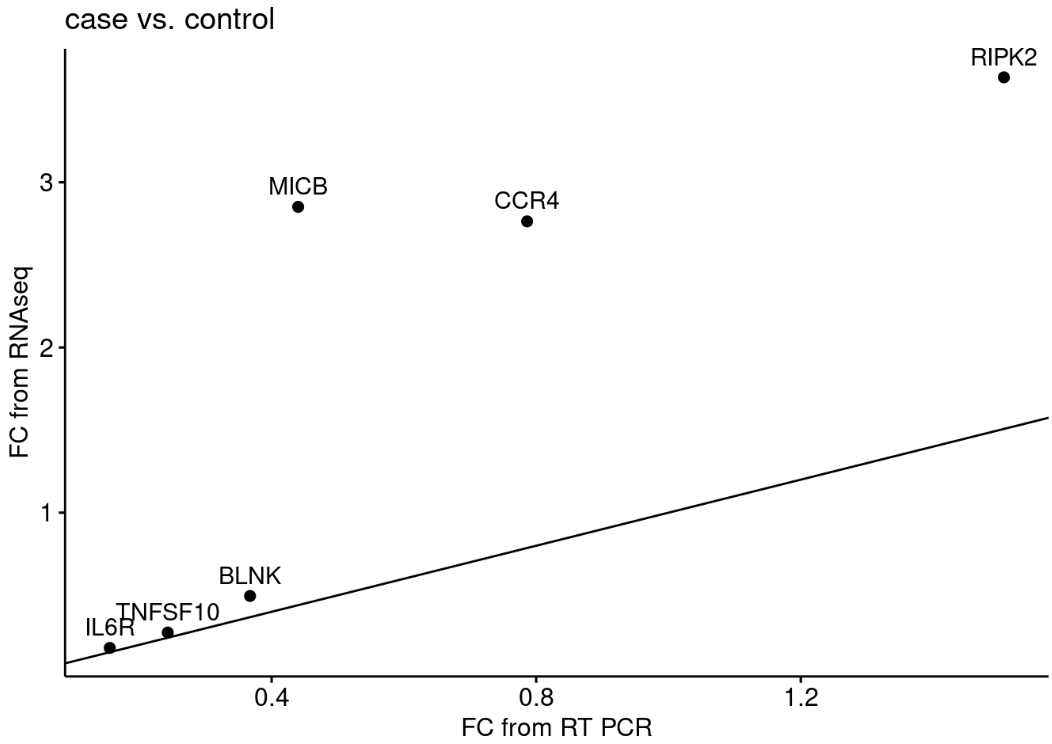

crossplot using FC from RT PCR based on all samples

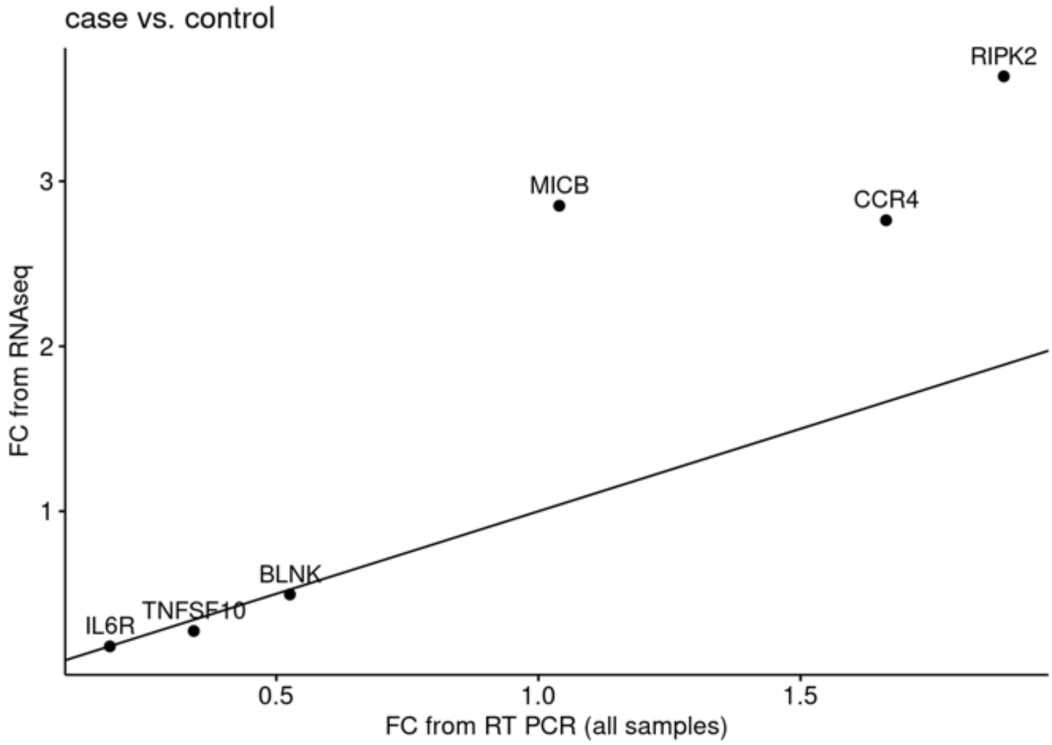

mts vs primary

```
[1] "33A" "34A" "39A" "28A" "40A" "46A"
```

crossplot of FC

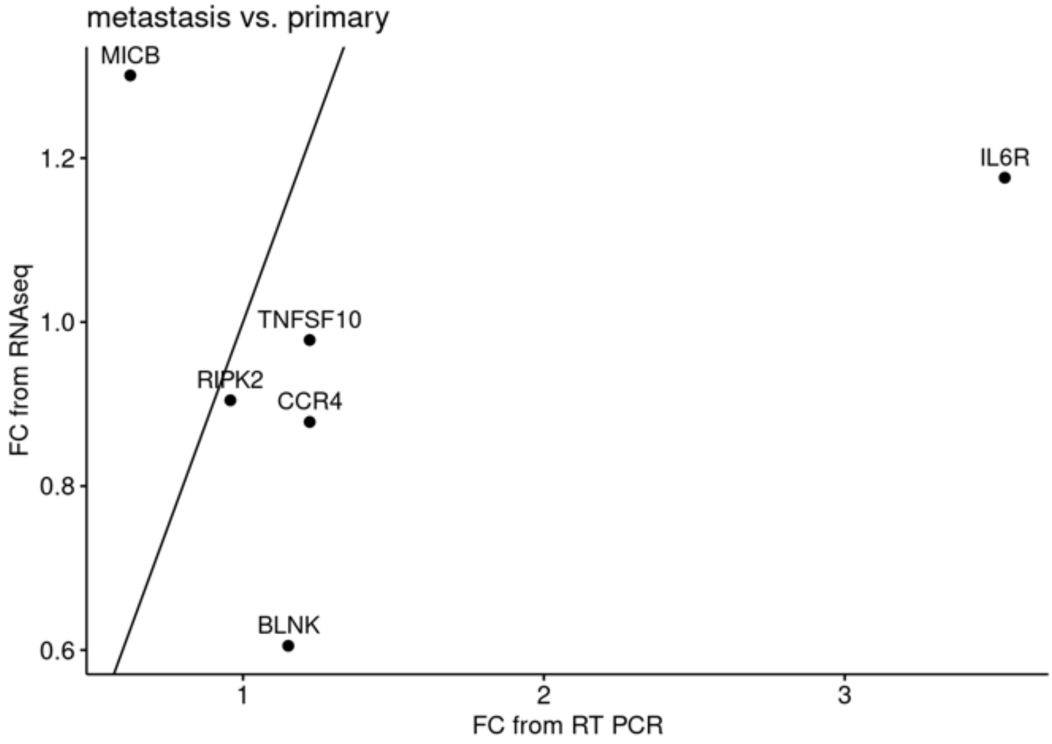

crossplot using FC from RT PCR based on all samples (28)

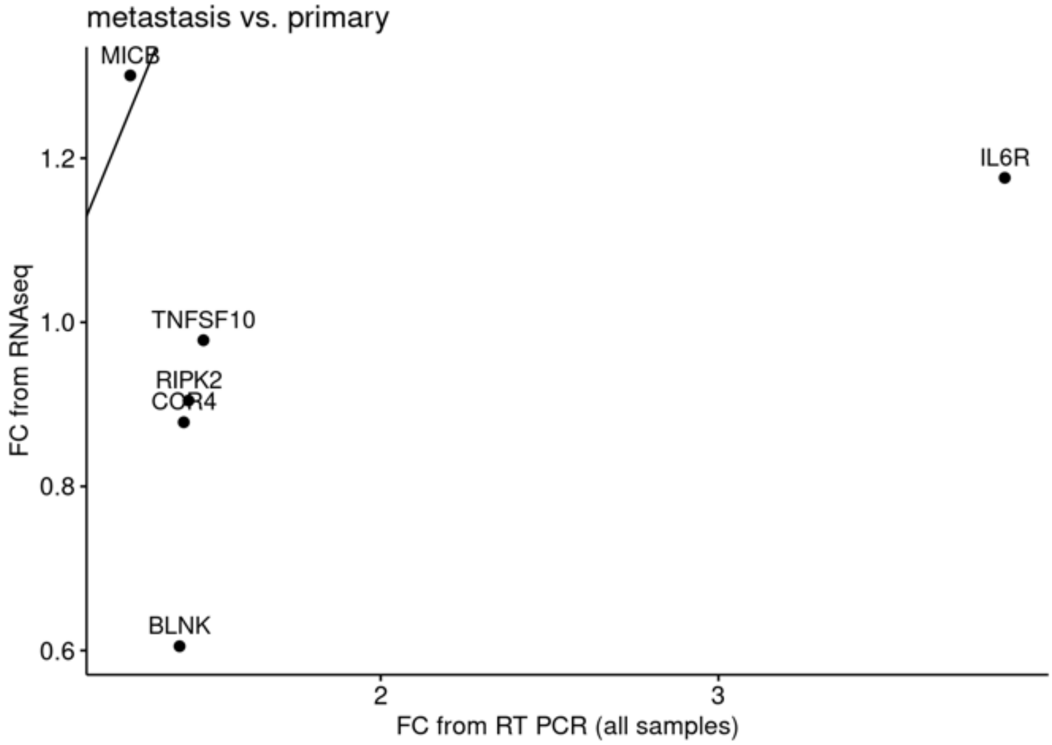

KRAS+ vs KRAS-

```
[1] "17A" "38A" "44A" "28A" "40A" "46A"
```

crossplot of FC

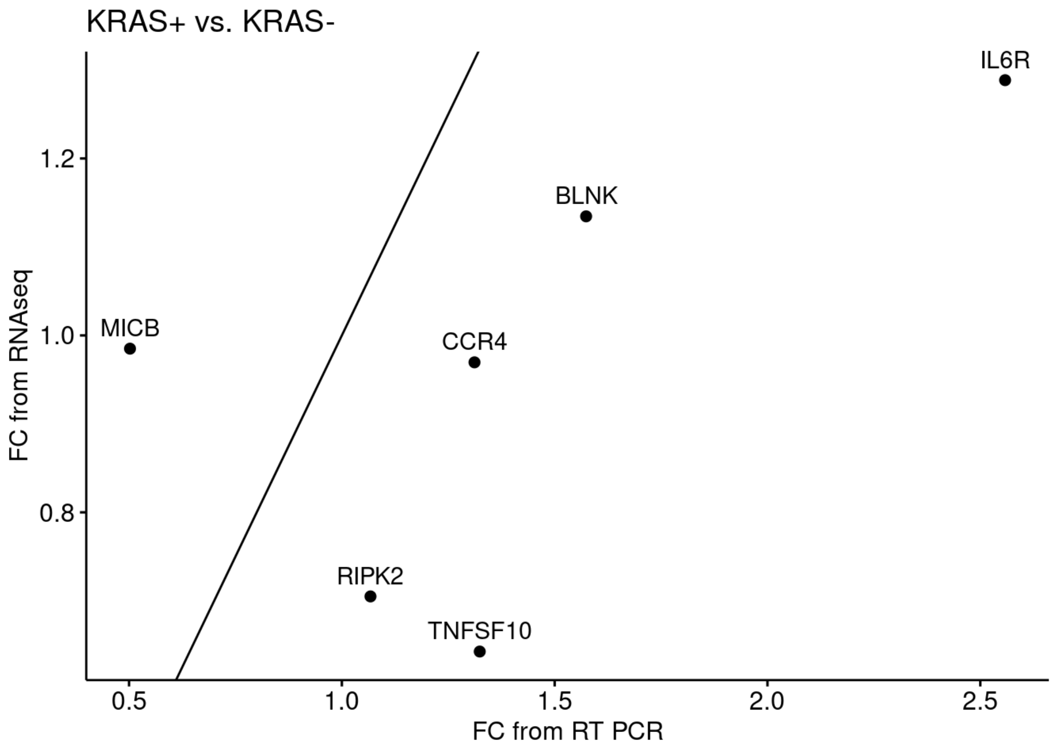

crossplot using FC from RT PCR based on all samples (31)

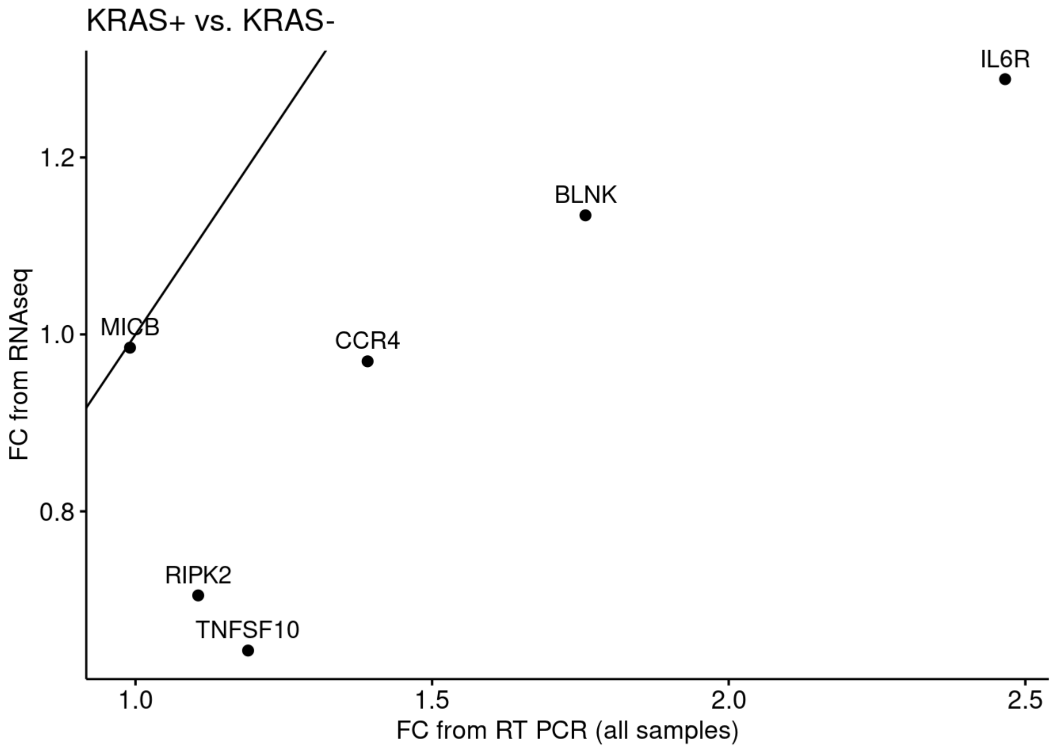

Notes

Fold Change (FC) from RNAseq was obtained by CLC Genomics Workbench. Statistical model used to obtain FC involved correction for batch effects.

FC for RT PCR was obtained the standard way. Since FC obtained from RNAseq is a single number (an average over the FC), the FC from RT PCR were averaged. The average was computed over the same samples that were processed by RNAseq. We have computed also the average of RT PCR-based FC over all samples.

The two sets of FC were plotted on a crossplot. The 45 degree line was added to the plot, to facilitate comparison of the two sets of FC.
