# Supplementary figures and images for: Differential gene expression of immunity and inflammation genes in colorectal cancer using targeted RNA sequencing
Source: Front Oncol. 2023 Oct 5;13:1206482. doi: 10.3389/fonc.2023.1206482 (PMC10586664; doi:10.3389/fonc.2023.1206482)

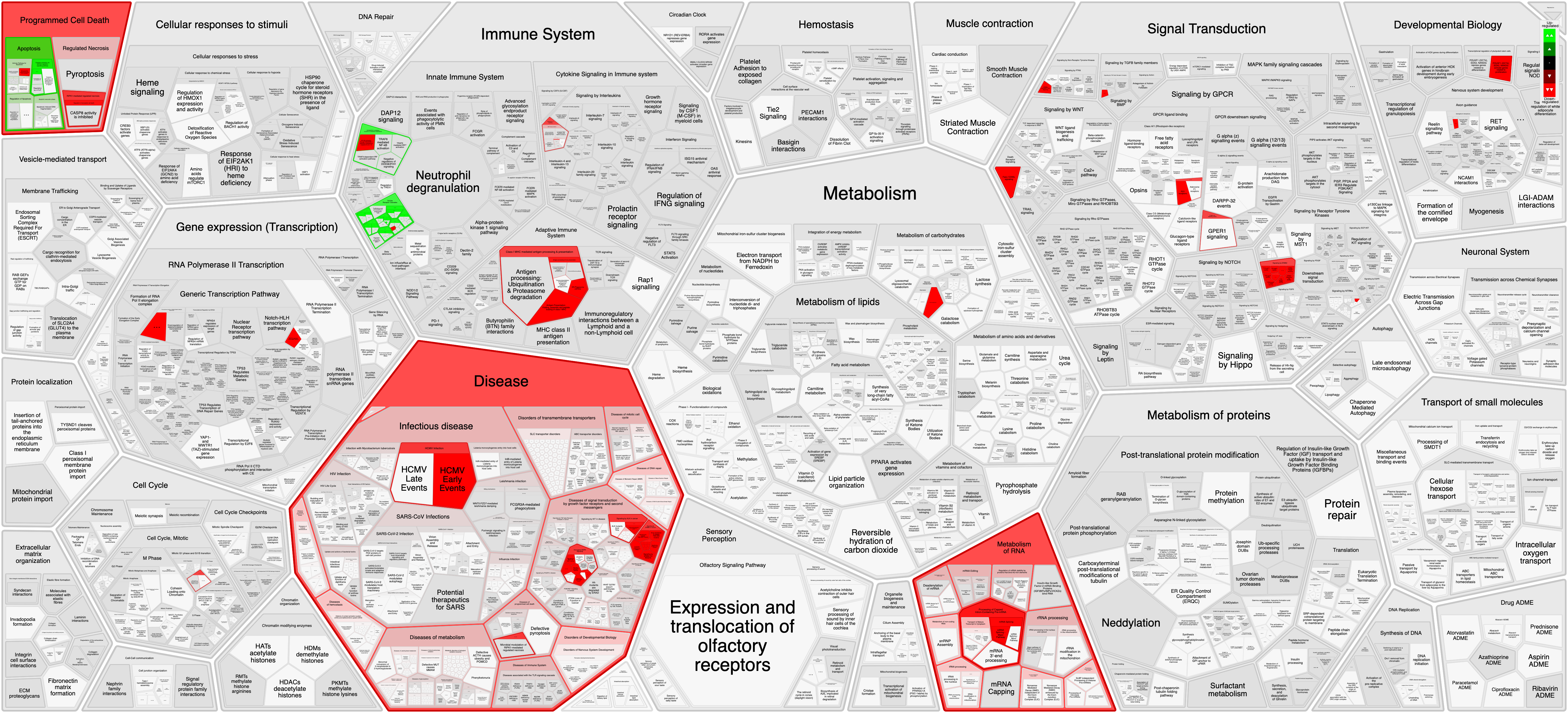

Supplement: Supplementary file 3 [file Image_1.jpeg]

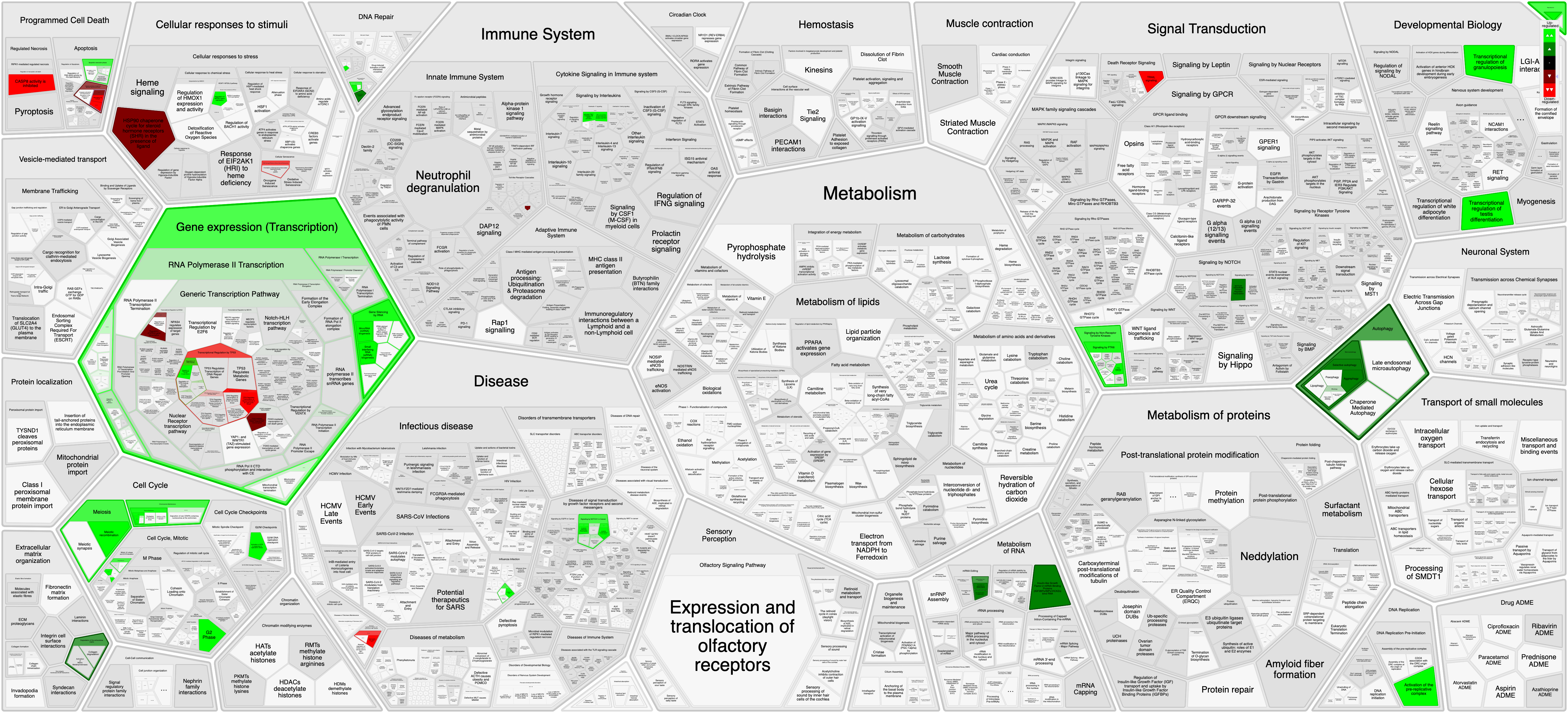

Supplement: Supplementary file 4 [file Image_2.jpeg]

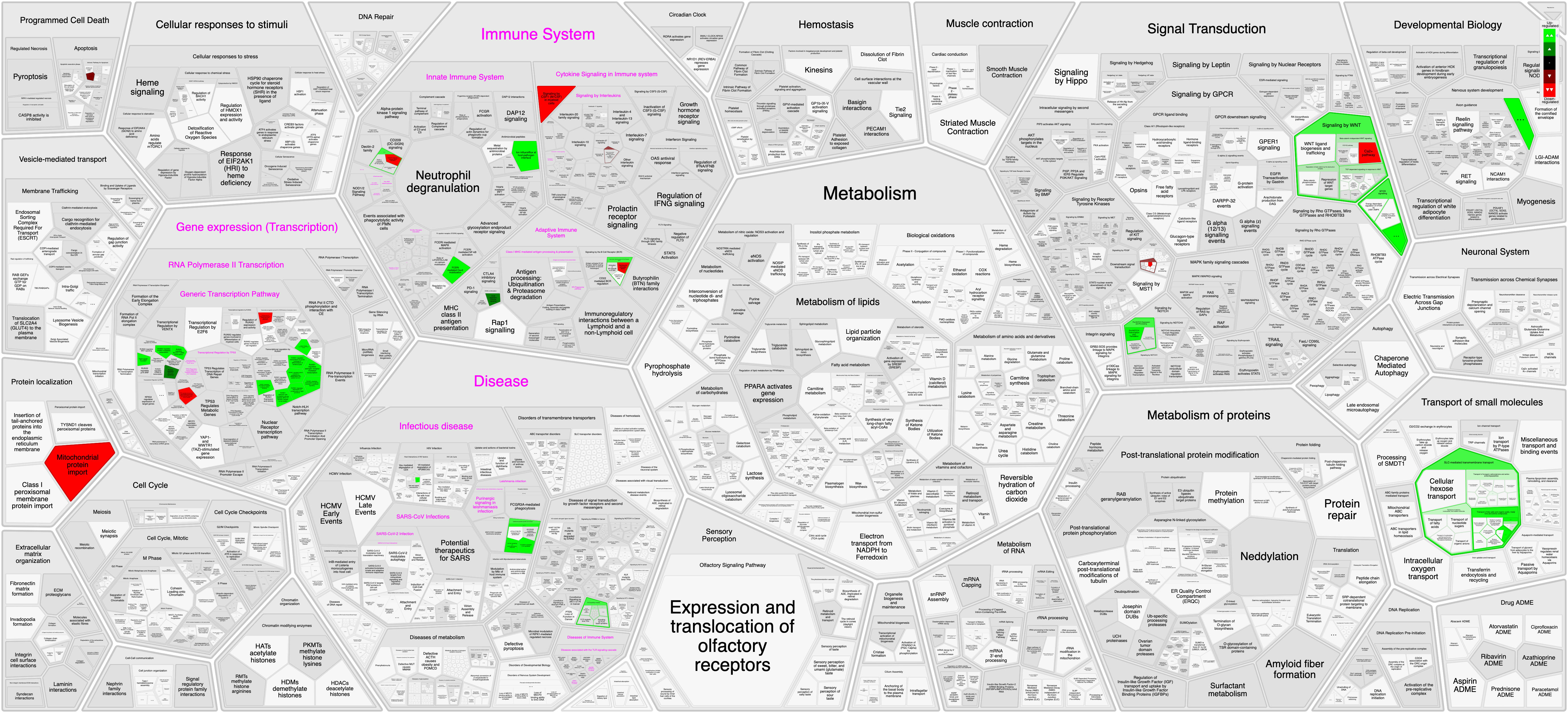

Supplement: Supplementary file 5 [file Image_3.jpeg]
